# Supplementary material for: Causal manipulation of self-other mergence in the dorsomedial prefrontal cortex
Source: Neuron. 2021 Jul 21;109(14):2353–2361.e11. doi: 10.1016/j.neuron.2021.05.027 (PMC8326319; doi:10.1016/j.neuron.2021.05.027)
Supplement: Document S1. Figures S1–S8 [file mmc1.pdf]

Neuron, Volume *109*

Supplemental information

Causal manipulation of self-other mergence

in the dorsomedial prefrontal cortex

Marco K. Wittmann, Nadescha Trudel, Hailey A. Trier, Miriam C. Klein-Flügge, Alejandra Sel, Lennart Verhagen, and Matthew F.S. Rushworth

Supplementary figures

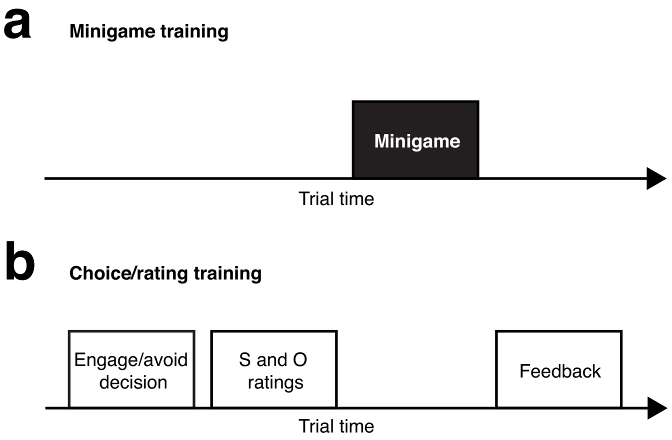

Figure S1. Instruction procedure. Related to Figure 1. The experimental instructions followed a precise schedule implemented according to our previous study (Wittmann et al., 2016). On the day of the 'taster session', when participants were introduced to TMS and their active motor thresholds were assessed, they also received full instructions for the experiment (~1 hour), which were briefly recapitulated on the day of the fMRI experiment. In the instructions, participants were told that they would play minigames during the experiment and their goal was to learn about their own and other players' performances and to make good decisions and ratings based on these pieces of information. It was emphasized that doing this would enable them to collect as many reward points as possible during the experiment and that they should aspire to do that. They were told that the performances of the other players that they would be paired with in the fMRI experiment was pre-recorded and they agreed that their own, fully anonymized performance data could be used for the same purpose in the future. Participants were instructed on the minigames, the ratings, and the engage/avoid decisions and performed example trials. The instructions were designed such that actual performance learning would only take place in the fMRI experiment to maximize learning effects in the scanner. (**a**) To still guarantee familiarity with the minigames, a written explanation of the two minigames was complemented with a short practice session, in which participants performed trials of each minigame. During those trials, the experimenter was present and made sure that participants understood the minigames. No explicit performance feedback was given on those trials to avoid performance learning. (**b**)To guarantee that participants understood the logic of the engage/avoid decision and the ratings, participants performed example trials that did not include minigames, but instead a placeholder screen. This allowed participants to adjust to the trial events and experience the reward outcomes of ratings and decisions. Importantly, the performance feedback on those trials followed no across-trial contingencies and consisted mostly of the highest or lowest performance feedback for the players. This was done to make these example trials very different from the trials experienced in the main experiment. Although participants could not learn anything during those trials and therefore could make no well-grounded decisions, they were asked to invent and verbalize reasons for their ratings and decisions so that the experimenter could make sure that they understood their logic (e.g. "This is a cooperate trial. I press the "engage" button, because although the threshold is high, I think we will perform very well. I rate myself and the other one positively, because I think we will both perform well..."). In sum, participants practiced all aspects of the experimental task, but in such a manner that they could not yet learn about their performance. Participants were told that their goal in the experiment was to collect as many points as possible and that points would be translated into monetary reward at the end of the experiment. It was emphasized that points could be earned by making good decisions and by providing accurate ratings of performance. Despite the substantial time needed for a thorough instruction, as in our previous study, most participants found the task intuitive and the behavioral data acquired in the experiment (see main text) confirmed that they understood the task.

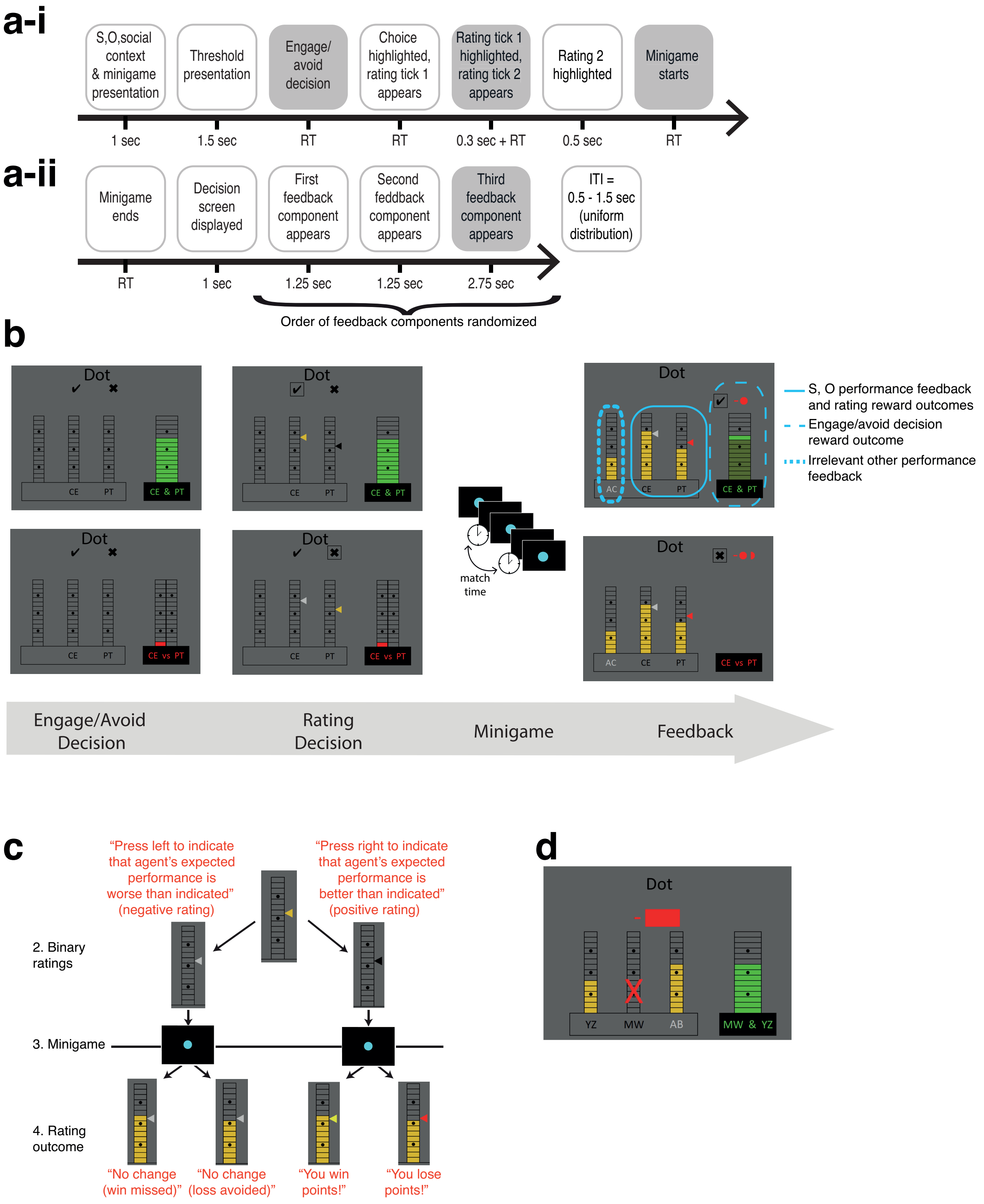

Figure S2. Trial structure, key events, rating rationale, and false starts. Related to Figure 1. (a) All events within a trial and their associated timings are depicted. Note that timing of the events is shown underneath the arrow and RT (reaction time) means that the respective step only ends when the participant makes a

response. Events highlighted in grey are depicted in more detail in (b) as they represent key events within the trial: engage/avoid decision, the rating decision, playing of the minigame and the feedback phase. The first part of a trial reveals the social context (competition or cooperation), participants then made an engage/avoid decision, and provided self (S) and relevant other (O) ratings **(a-i).** The feedback phase shows performances of all players during the minigame and possible points gained or lost from engage/avoid decisions and rating decisions. **(a-ii)**. **(b)** For illustration purposes, the same performance feedback is shown in a cooperative context (upper rows) and a competitive context (lower rows). At the beginning of a trial S (initials of the participant, middle position), O (initials of one of the two other players relevant for the current trial, left or right position), social context (cooperation or competition) and type of upcoming minigame ("Dot" indicates the dot task; see supplementary figure 3) is presented. The initials of the irrelevant other player are not shown, and the social context is color-coded as green for cooperation and red for competition. These colors are also used to indicate the threshold of the current trial (thresholds are shown as bars on the very right). In these examples, the choices made (see highlighted tick and cross in the first panel) are to engage in cooperation and to avoid competition. The choices are arbitrarily picked for illustration here, but in the experiment the decision to engage in cooperation would indicate the expectation that S and O will, together, perform better than the threshold of 10; the avoidance of competition would indicate that the participant does not expect to perform at least one point better than the O. After the engage/avoid choices, binary ratings of S and O take place (second panel) in randomized order. Once a rating has been made, the tick turns from yellow to either grey or black depending on whether one expects the selected player to perform respectively better or worse compared to the level indicated by the tick. In these examples, O rating occurred first in the cooperation trial and S rating occurred first in the competition trial (ratings have already been made and are therefore shown in grey/black). These ratings indicate that the participant expects to perform worse than 10.5, while the O is expected to perform better than 8.5 (see panel (c) for more information on color coding of rating ticks). Note that these ratings are consistent with the engage/avoid choices shown in the two examples. In the feedback phase (third panel), the previous screen from before the minigame reappears (see a-ii). However, the right side of the screen showing the cooperation/competition threshold is occluded if a participant had chosen to avoid cooperation/competition in the previous decision phase of the trial. This means that in this example trial the threshold is only shown again in the cooperate trial (because the participant actually decided to cooperate) and not in the compete trial (because in this example trial the participant refrained from competing). In other words, the repeated presentation of the threshold is not a feature of the social context, but entirely a consequence of the engage/avoid choice made. Subsequently, three feedback components appear in randomized order to control for sequence effects (see legend on the right-hand side). In cooperation, the choice payoff is -1 (red coin above threshold), because the average performance is 9 while the threshold is 10. In competition, the choice payoff is -1.5 which is due to chance (payoff from avoid choices is +1.5 or -1.5 with a 50/50 probability) and independent of performance feedback. Note that the participant would have earned a payoff of 3 had the engage choice been taken (performance feedback difference of +4 minus threshold of +1). Note that, overall, the magnitude of the rating payoff is marginal compared to the engage/avoid choice payoff. **(c)** Rationale of binary ratings. Before the minigame, participants indicated for S and O either a positive or a negative rating reflecting the expectation that the player would surpass or fall below a given rating marker. The color change of the rating marker was indicative of the choice made; the rating marker turned black for a positive rating and grey for a negative rating. A positive rating led to a win or loss of 0.25 points depending on subsequent performance feedback. A negative rating led to no change in the points count independent of performance feedback. Therefore, making a correct negative rating was associated with a benefit of avoiding losing points while making a correct positive rating was associated with a benefit of winning 0.25 points. Note that in panel (a-ii), the performance and rating feedback indicate an incorrect negative rating (missed win) for S and an incorrect positive rating (loss) for O. Red text in quotes is taken from the participants' instructions where a similar illustration was used. **(d)** The screenshot shows the feedback screen of a false start trial. In false start trials, the true performance of the participant in the minigame was below a predetermined threshold for acceptable performances. False start trials were a case of veridical performance feedback to ensure that performance feedback in general was believable. Participants incurred a loss of 3 points (indicated by the red bar) on those trials and no points could be won by the decision or the ratings. The feedback phase in false start trials was not analyzed and no prediction error for S was calculated on those trials (reward prediction error and prediction error for others were calculated as normal).

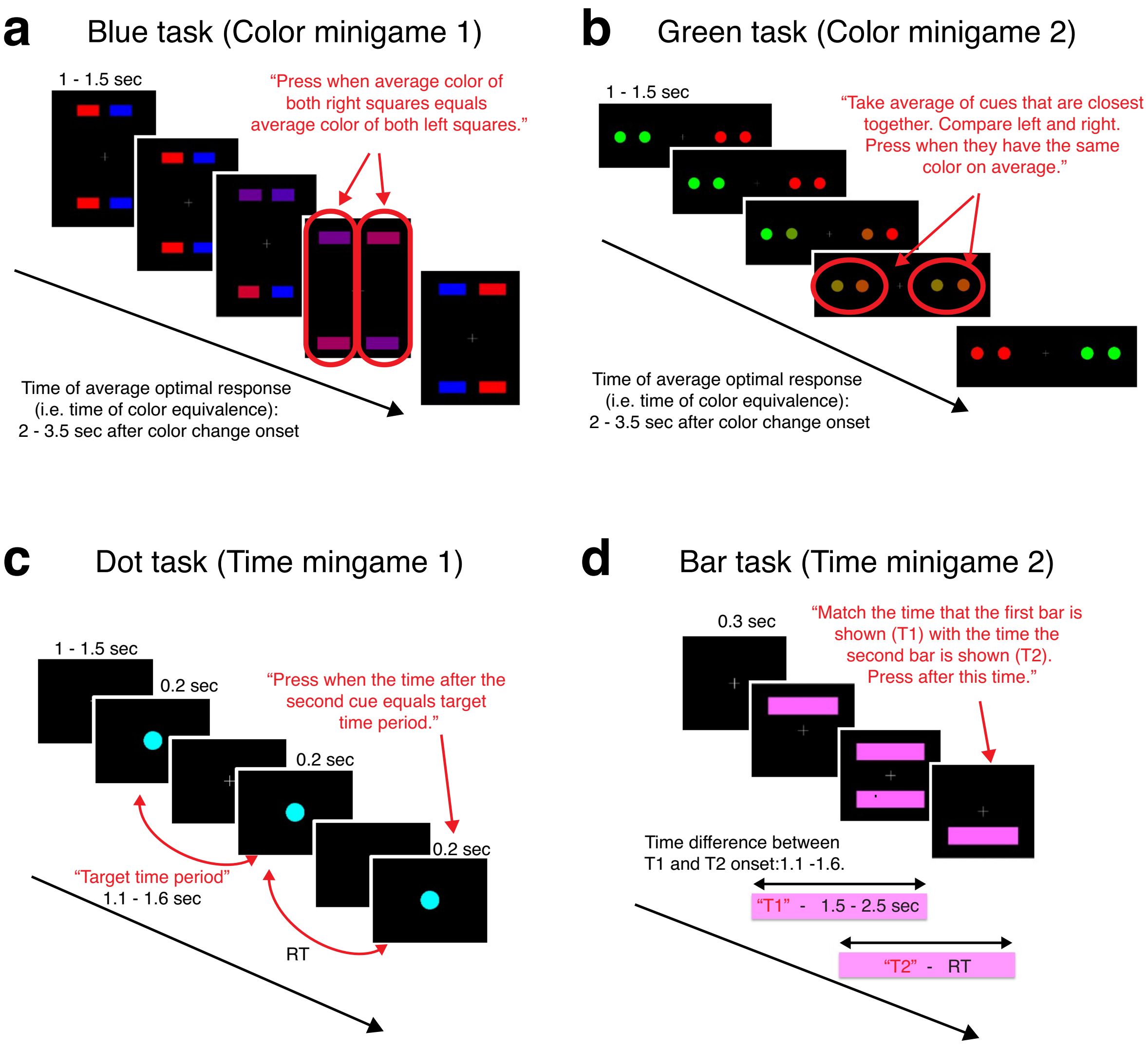

Figure S3. Minigame description. Related to Figure 1. We used two pairs of minigames in the experiment ('color minigames' and 'time minigames'), each assigned to one fMRI block. The order of the types of minigames was counter-balanced across participants (color minigames were assigned to the first session for one half of participants and to the second session for the other half) and this was orthogonal to the application of TMS. The minigames were necessary prerequisites for administering performance feedback on every trial, which was in fact pre-determined according to a finely balanced and decorrelated schedule. However, exceptions from this pre-determined schedule existed and constituted cases of veridical performance feedback; this was an additional measure to ensure that performance feedback was believable. These exceptions were 'false start' trials, in which the true performance was worse than a given performance threshold, and this then led to a feedback penalty (see section *Experimental Paradigm* of the supplementary text document). Minigames were short reaction-time based tasks and we designed them to be relatively non-transparent in the sense that the experience of performing the minigames was not very informative for estimating one's ability compared to the explicit performance feedback that was given to the participants. Also, we varied the timing parameters of the minigames to make it harder to compare true performance across different trials of a minigame and to ensure a temporal jitter between decision and outcome phases. The two time minigames were relatively similar to each other (participants estimated a target time period) and so were the two color minigames (participants estimated color equivalences). In consequence, the two fMRI sessions were framed to the participants as measuring very different aspects of cognitive performance. Generating this impression was important, because we used the same performance schedule for both fMRI sessions (see *Experimental task* section in Methods; Supplementary Fig.2). This meant that we could investigate and compare the behavioral and neural mechanisms underlying learning with the same performance schedule both with and without the application of cTBS. The framing in terms of very different types of minigames prevented participants from noticing this similarity and in fact none of the participants

expressed any suspicion spontaneously or after being questioned. Each minigame was performed with one response button press with the right index finger and the approximate time for each of the four minigames was similar (see figure for timing parameters). As in our previous study, participants performed all four tasks very well(Wittmann et al., 2016). We calculated an index of true performance in the tasks in the same way as in our previous work(Wittmann et al., 2016). However, this performance index was not used further when analyzing behavioral data; it was only used to determine thresholds for the above-mentioned 'false start' trials (see *Experimental task* section in Methods; Supplementary Fig.2), which was based on pilot experiments. Note again, that except for false start trials, the performance feedback was unrelated to this true performance measure. Note that the red text in the panels was used to explain the task to participants, who saw very similar illustrations of the task in the instructions. Black text supplements timing information. All varying timings were picked from a uniform distribution. **(a,b)** In the color minigames, two pairs of cues appeared on the screen and after an initial stable period, they began to change color until the colors fully reversed. The two color minigames, the blue task (panel a) and the green task (panel b), differed with respect to the colors used and the spatial organization of the colored cues on the screen. The underlying principle was the same however: Participants indicated with a button press when the colors of two of the cues reached the same average color as the two other cues that changed color in the opposite direction (red boxes indicate which cues were compared). This point of true color equivalence was reached after 2 – 3.5 seconds. **(c,d)** In the two time minigames, participants replicated a given time interval with the goal of matching a target time. In the dot task, this target time is the time between two blue dots that appear briefly on the screen in sequence. In the bar task, the target time is the time that a bar is initially presented on the screen and the goal is to replicate the target time for a second bar that occurs on the screen after a short delay. Target times were 1.1 – 1.6 seconds for the dot task and 1.5 – 2.5 seconds for the bar task. Although these target times were different, the overall time spent on both minigames was similar.

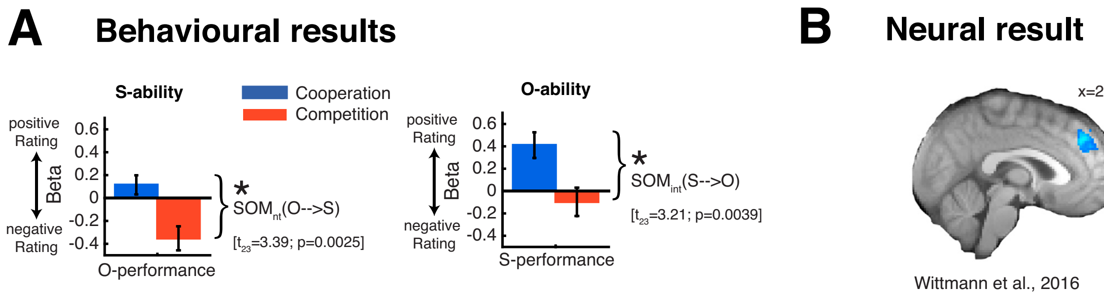

Figure S4. Results of previous report of self-other-mergence (Wittmann et al., 2016). Related to Figure 3. **(A)** Previously, we applied the same statistical analyses to the rating data for S-ability and O-ability and found significant SOM_{int} in both cases (blue = cooperative trials, red = competitive trials). Note that, as done here, significance testing was performed on the full GLM as reported in Supplementary Fig.5A. **(B)** Previously, we identified dmPFC area 9 using the contrast O-performance (shown in blue). In ROI analyses performed on the peak coordinates of this contrast, we found a significant neural SOM_{int}(S-->O) effect that correlated with the behavioral neural SOM_{int}(S-->O) effect. Therefore, we used the peak coordinates of the contrast shown in panel B as the target location for our dmPFC-cTBS group. Data are represented as mean ± SEM.

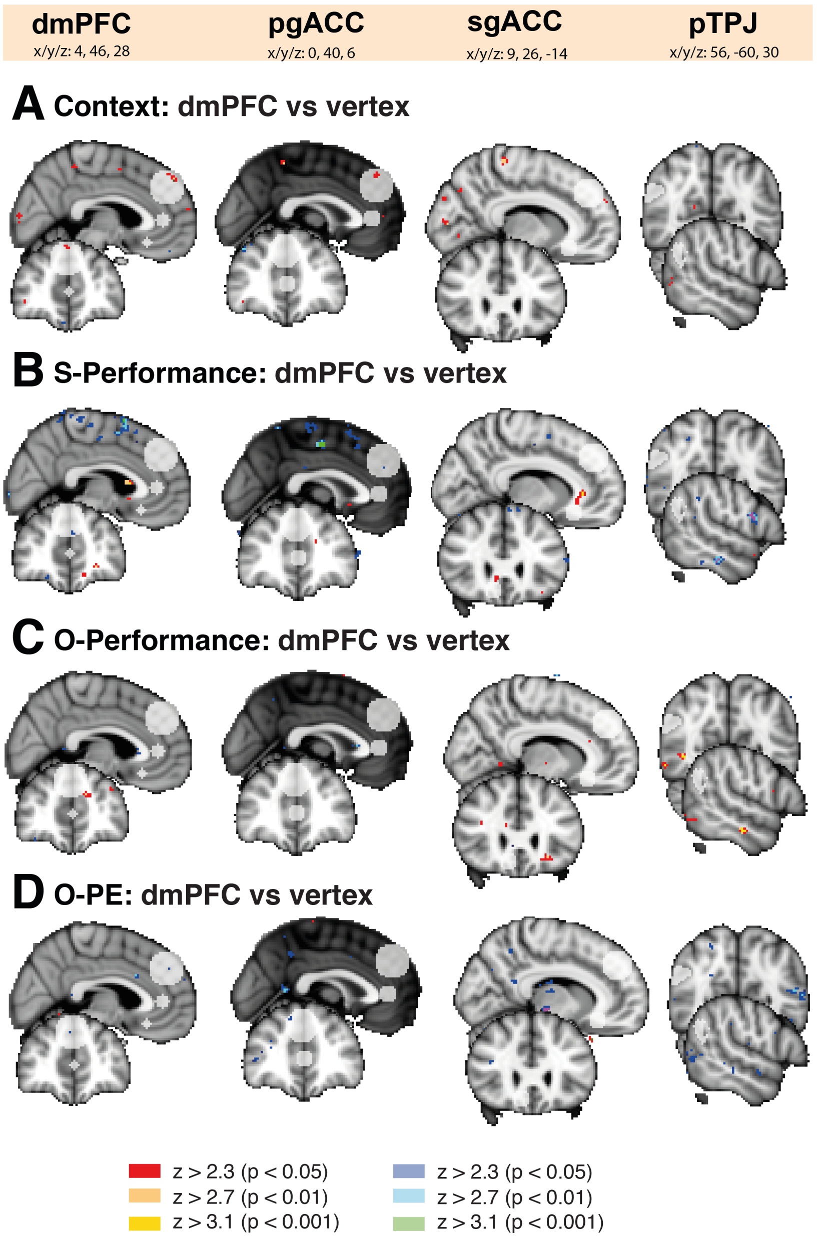

Figure S5. Additional neural effects of cTBS including sub-threshold activations. Related to Figures 5 and 6. Brain-wide effects are shown for the contrasts related to Fig.3A,B. Colors present uncorrected z-maps thresholded at z>3.1, z>2.7 and z>2.3 (red and blue represents respectively positive and negative activation). For transparency, we are showing additional variables that might potentially have been affected by dmPFC stimulation. As in Fig.6 we overlay additional ROIs in areas relevant to social cognition (pgACC, sgACC and pTPJ; see Fig.6 and main text for details). Modulation of activation is shown as a difference between cTBS and no-cTBS sessions in the dmPFC group compared to the control group. We do not observe modulation of activation as a result of stimulation (neither whole-brain nor in the ROIs) for **(A)** context (cooperation=1, competition=-1), **(B)** S-performance (performance history of Self), **(C)** O-performance (performance history of Other), or **(D)** O-PE (Prediction error associated with other's performance). These results show that dmPFC stimulation was specific to the SOM_{int}(S-->O) effects reported in the main text. In relation to the S-performance effect shown in panel B and our finding that S-performance was significantly changed during competition, we considered whether the S-performance effect in competition was related to the strength of SOM. However, there was no correlation between the neural effect of S-performance in no-cTBS during competition (collapsed over both groups; ROI taken from Fig.5C) and SOM_{int}(S-->O) (r= -0.140; p = 0.302) during no-cTBS.

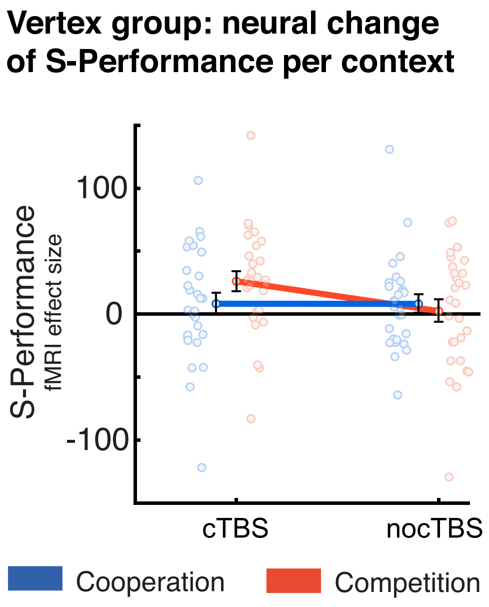

Supplementary Figure 6. Neural effects of S-performance in the vertex control group. Related to Figures 5 and 6. The panel shows the same regressor as main text Fig.5D, performed on fMRI data from the same ROI as in Fig.5D, but for the vertex control group. Data are represented as mean ± SEM.

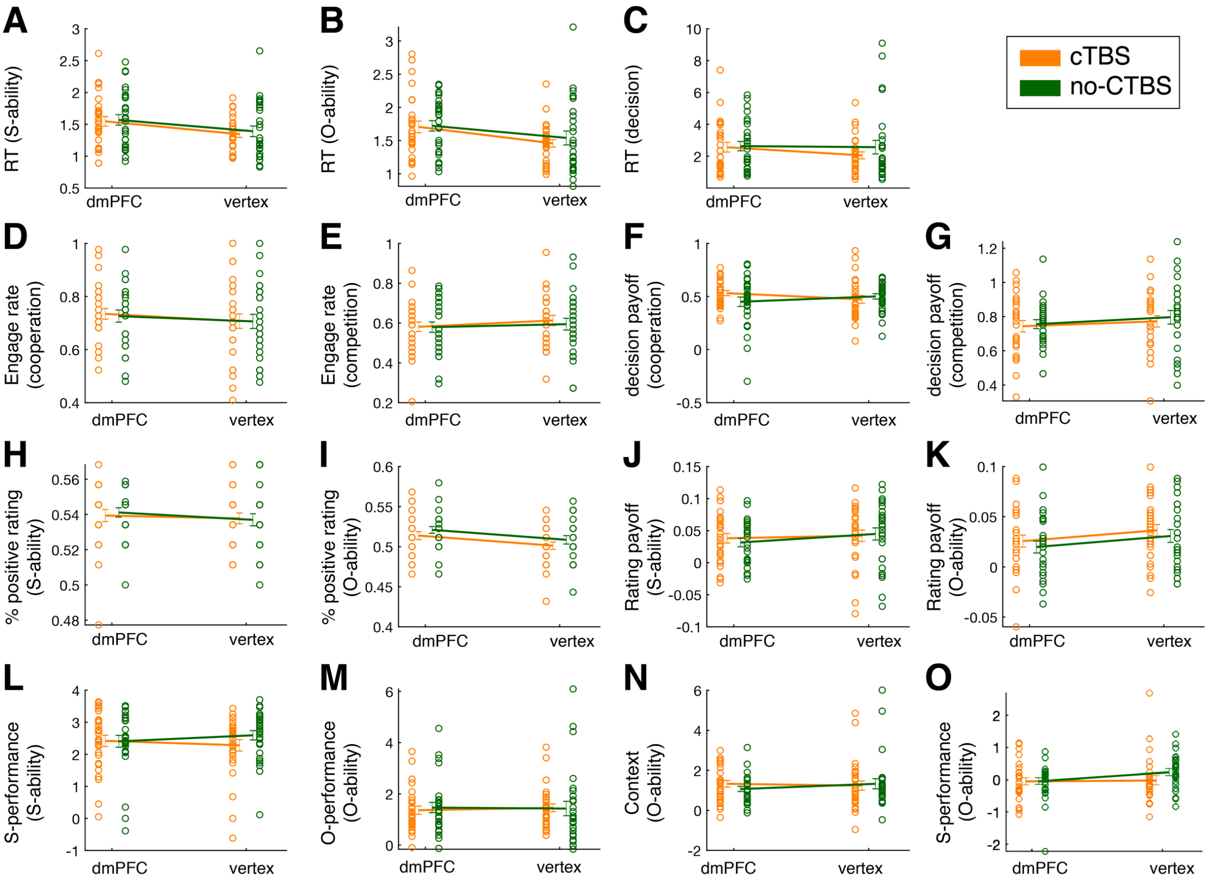

Figure S7. cTBS over dmPFC does not impact general task performance. Related to Figures 7. We conducted several behavioral control analyses that targeted different task-related variables that might change as a function of the application of cTBS over dmPFC. As for our critical statistical test of interest, the cTBS induced change of SOM_{int}(S-->O), we calculated a mixed-effects analysis of variance to test for a dmPFC/vertex by cTBS/no-cTBS interaction effect. Despite considering a broad range of task variables, we found no significant interaction effects, suggesting that our causal manipulation targeting dmPFC specifically impacted SOM_{int}(S-->O). **(A,B,C)** First, we considered reaction times related to the S-ability and O-ability ratings as well as to the engage/avoid decision. Such reaction time effects might reveal broad changes in the time necessary to compute information for oneself, the other player, or to combine this information flexibly towards a decision. Hence, analyses of reaction time might reveal specific deficits in one or several of these domains. We found no significant interaction effect either for the reaction times for S-ability-rating ($F(1,54) = 0.042$, $p = 0.839$), O-ability-rating $F(1,54) = 0.260$, $p = 0.612$), or for the engage/avoid decision $F(1,54) = 1.670$, $p = 0.202$. **(D,E,F,G)** Next, we considered whether the general ability to make decisions in cooperative or competitive contexts was impacted by the application of cTBS. Decisions require an integration of self-and other-related performance estimates and might be impacted differentially based on whether the context required participants to combine performance estimates during cooperation or to contrast performance estimates during competition. Therefore, we examined whether the frequency of making an engage choice was altered by the application of cTBS over dmPFC. There were no significant interaction effects for the rate of engage choices either in cooperation ($F(1,54) = 0.062$, $p = 0.804$), or in competition ($F(1,54) = 0.334$, $p = 0.566$). In addition, the payoff that resulted from these decisions did not change as a function of the cTBS application either in cooperation ($F(1,54) = 2.872$, $p = 0.096$) or competition ($F(1,54) = 0.030$, $p = 0.863$). **(H,I,J,K)** After this, we considered whether stimulating dmPFC might have affected performance estimation for one of the players in some more general way as opposed to in a manner that was specifically related to self-other-mergence. We compared the rate at which a player was estimated as better than indicated by the rating tick (see Supplementary Fig.2 for details of the rationale behind the ability rating). However, this measure of rating performance was not impacted by the cTBS stimulation either for S-ability ratings ($F(1,54) = 0.339$, $p = 0.563$) or for O-ability ratings ($F(1,54) = 0.001$, $p = 0.985$). In addition, the payoff received for S-ability ratings ($F(1,54) = 1.121$, $p = 0.294$) and O-ability ratings ($F(1,54) = 0.001$, $p = 0.979$) was not significantly impacted by the cTBS. The absence of such general performance impairments further suggests that our causal manipulation impacted a very specific aspect of self and other-related performance estimation. **(L,M,N)** We went on to perform an even more

sensitive test to assess whether cTBS over dmPFC induced deficits in “appropriate” S-ability or O-ability estimation – the degree to which ability estimates reflect performance by the same person (i.e. appropriate estimates of O-ability should be based on O-performance and appropriate estimates of S-ability should be based on S-performance). Specifically, we extracted the S-performance effect from the S-ability GLM and compared it across stimulation conditions and groups. We performed an analogous comparison for the O-performance effects in the O-ability GLM. Both of these effects assess how much estimates of ability are based on the previous history of performance of the same player. We believe that this is the most sensitive measure of appropriate ability performance learning in our current set of analyses. We performed this test by comparing effect sizes from our main GLMs of interest predicting S-ability and O-ability (Figs.4,7). Note that we used the variance-weighted beta weights (Matlab’s stats.t object) for the S-ability GLM to approximate the effect sizes because of difficulties in estimate effect sizes for all participants as described in the methods. The O-ability GLM employed standard beta weights as proxies for the effect sizes. However, we found no cTBS induced changes in appropriate ability estimation either for S-ability ($F(1,54) = 1.422$, $p = 0.238$) or for O-ability ($F(1,54) = 0.094$, $p = 0.760$). **(N)** Next, we considered whether cTBS over dmPFC might have affect a more non-specific bias in the estimation of the other player’s performance. As we have shown in Fig.4 (see main text), participants showed an other-specific optimism bias in performance estimation. They evaluated the other player more positively in cooperation than during competition. Importantly, this bias is present in addition to SOM_{int} and, in contrast to SOM_{int}, it is unrelated to the specific levels of performance of the irrelevant player. However, the optimism bias in performance estimation (*Context* effect in O-ability-ratings) was not altered by the cTBS ($F(1,54) = 0.975$, $p = 0.328$). Together, these results suggest that cTBS over dmPFC specifically altered only SOM_{int}(S-->O). It did not affect more general measure of task performance including choice frequencies and payoffs during decision, Self-rating and Other-rating. Neither did it induce a general deficit in performance tracking for one of the agents, or changed the optimism bias during the O-ability estimation. Instead, as shown in Fig.7, cTBS over dmPFC specifically affected the degree to which O-ability was referenced to one’s own performance in a context-dependent manner. **(O)** Finally, we considered whether cTBS might have impacted on S-performance during the estimation of O-ability. S-performance is tightly related to our key effect of interest, SOM_{int}(S-->O), which is the effect of “S-performance x Context” in the estimation of O-ability. Significant effects of S-performance here indicate that cTBS-induced changes in SOM occur mostly in one of the two social contexts, cooperation or competition. However, we did not find a significant main effect of S-performance on O-ability estimation ($F(1,54) = 1.712$, $p = 0.196$) suggesting that dmPFC cTBS does not result in uniformly increased or decreased effects of S-performance on O-ability and therefore also does not suggest that cTBS-induced changes on SOMint(S-->O) are mainly driven by one of the two social contexts. Data are represented as mean $\pm$ SEM.

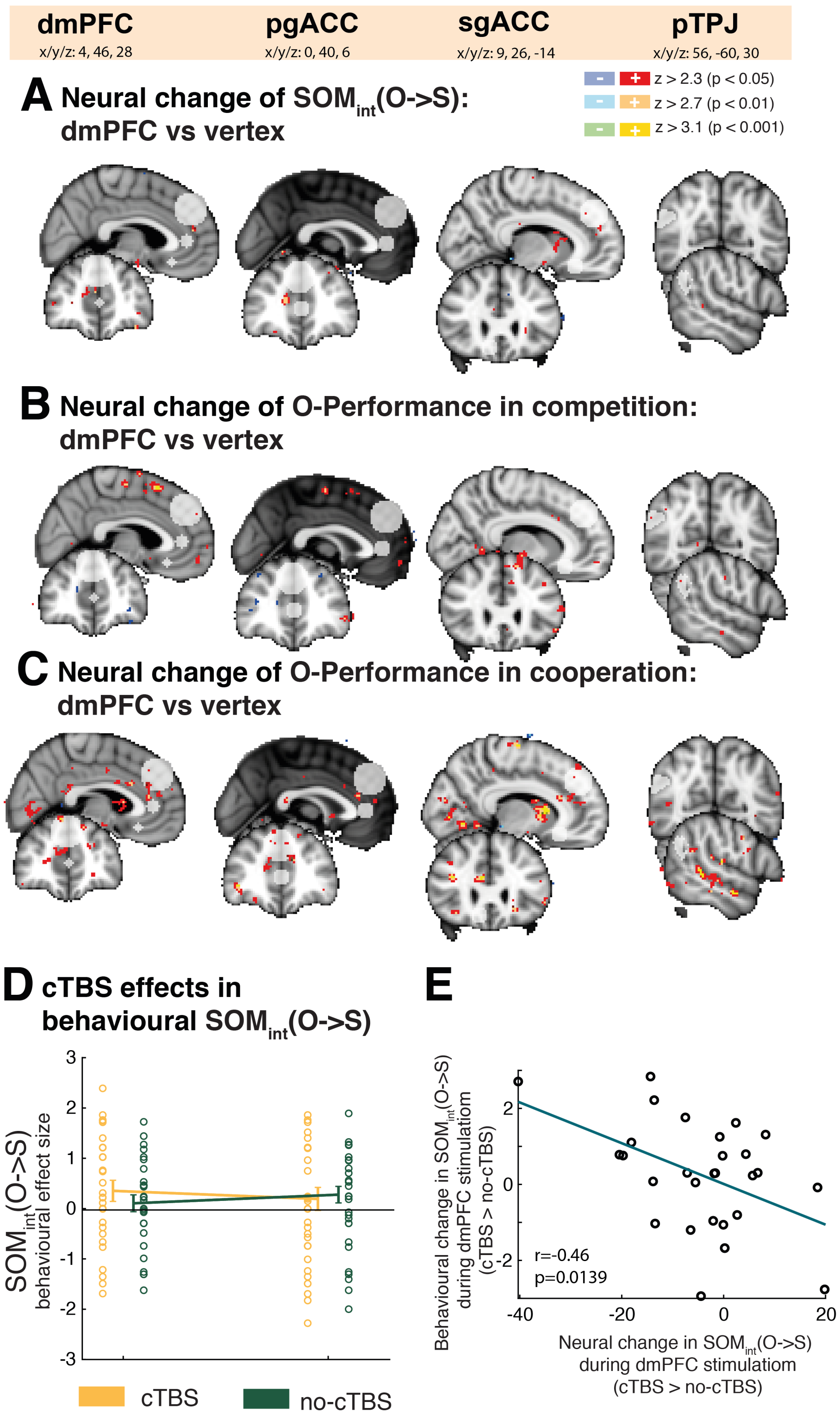

Figure S8. cTBS effects on SOM_{int}(O-->S). Related to Figure 5,6 and 7. In our previous work (Wittmann et al., 2016), we found that signals in dmPFC were correlated with self-other-mergence in multiple ways suggesting that it encodes reciprocal influences of our own performance estimates on estimates of another person and vice versa. However, of the two self-other-mergence effects we discovered in behavior, only SOM_{int}(S-->O) was directly correlated with neural activity in dmPFC. For this reason, in the main text, we focus on this direction of

influence instead of SOM_{int}(O-->S). To complement our results in the main text, in this supplementary figure, we examine the effects of cTBS on SOM_{int}(O-->S) following the same analysis steps as for our main analyses. **(A,B,C)** These panels show a series of whole brain analyses examining SOM_{int}(O-->S) related effects in the same region of interest in dmPFC. As in Fig.6 in the main text we overlay additional ROIs in areas relevant to social cognition (pgACC, sgACC and pTPJ; see Fig.6 and main text for details). We considered whether cTBS might have an effect on SOM_{int}(O-->S) (i.e. the variable *O-performance x Context,* see Methods; panel A). In addition, as we had observed that the effects of cTBS on SOM_{int}(S-->O) were particularly pronounced in the competition condition, we also separated SOM_{int}(O-->S) into its component parts: *O-performance* during competitive trials (panel B) and *O-performance* in cooperative trials (panel C). For all three contrast, we assessed cTBS-induced neural effects as the difference between cTBS and no-cTBS condition for dmPFC stimulation compared to the same difference for vertex control group. This stringent comparison ensured that we could isolate neural effects that specifically emerged as a consequence of causally manipulating activity in dmPFC. More formally, the contrasts show the effects of "dmPFC (cTBS – no-cTBS) > vertex(cTBS – no-cTBS)" for all three variables of interest. However, we found no ROI corrected or whole-brain corrected results for any of the three contrasts neither in dmPFC nor in any of the other social cognition-related ROIs. Effects are shown at uncorrected threshold levels for illustration (blue and red colors respectively, represent negative and positive activations). **(D)** Despite the absence of neural changes in SOM_{int}(O-->S), we examined whether the cTBS on dmPFC might have altered the behavioral effect of SOM_{int}(O-->S). We used the complementary GLM to the one for SOM_{int}(S-->O) (see main text and Fig.4, Methods) and examined the *O-performance x Context* variable. We used the t-stats for inference (see explanation in main text and Fig.4). We predicted that cTBS on dmPFC should have a similar effect on SOM_{int}(O-->S) as it had for SOM_{int}(S-->O), namely an increase in self-other-mergence after disruption of dmPFC activity with cTBS. Although the direction of effects that we found is consistent with this hypothesis, the relevant interaction between group (dmPFC/vertex) and stimulation (cTBS/no-cTBS) is not significant (2-way mixed effects ANOVA ($F(1,54) = 0.976$, $p = 0.328$). Therefore, consistent with the absence of neural changes in SOM_{int}(O-->S), we also did not find behavioral changes in SOM_{int}(O-->S) induced by cTBS over dmpFC. **(F)** Finally, in the absence of changes in neural or behavioral SOM_{int}(O-->S) in our sample as a whole, we considered the possibility that cTBS might have disrupted the neural representation of SOM_{int}(O-->S) more in some participants than in others and that these participants might in consequence display increased behavioral SOM_{int}(O-->S). We used a cluster-shaped mask of the effect shown in Fig.5B as region of interest, because the corresponding neural changes were the strongest cTBS induced effect in our data set. We reasoned that cTBS might have the strongest effects on SOM_{int}(O-->S) in this part of the brain. Note that this correlation analysis is statistically independent of the ROI selection because it examines individual variation in a variable unrelated to the ROI selection. From this ROI, we extracted the second level contrast maps (cTBS > no-cTBS) (see Methods for more information on the fMRI analysis pipeline). Indeed, we found a correlation between the neural change of SOM_{int}(O-->S) in the dmPFC group and their change in behavioral SOM_{int}(O-->S): Participants with a stronger decrease in neural SOM_{int}(O-->S) exhibited a stronger increase in behavioral SOM_{int}(O-->S) ($r=-0.46$; $p=0.0139$). In summary, these results show that both neural and behavioral SOM_{int}(O-->S) are not as strongly impacted as SOM_{int}(S-->O) by the application of cTBS over dmPFC. However, the correlation between neural and behavioral SOM_{int}(O-->S) effects across participants suggests that dmPFC carries information about self-other-mergence also in the direction other-to-self (Wittmann et al., 2016) – variation in neural signals predict the degree by which individuals are influenced by others in their self-assessments. Our results suggest that dmPFC is part of a wider neural network computing the relationships between self and other performance in both directions of influence. However, in contrast to the causal importance of dmPFC for SOM_{int}(S-->O), SOM_{int}(O-->S) might be controlled by a tightly connected brain region that shares information about the SOM_{int}(O-->S) with dmPFC. Data are represented as mean $\pm$ SEM.
