# Supplementary material for: Causal manipulation of self-other mergence in the dorsomedial prefrontal cortex
Source: Neuron. 2021 Jul 21;109(14):2353–2361.e11. doi: 10.1016/j.neuron.2021.05.027 (PMC8326319; doi:10.1016/j.neuron.2021.05.027)
Supplement: Document S2. Article plus supplemental information [file mmc6.pdf]

Neuron

Article

Causal manipulation of self-other mergence in the dorsomedial prefrontal cortex

Highlights

- During self-other mergence (SOM), people confuse one's own with another's performance
- Brain stimulation over dorsomedial prefrontal cortex (dmPFC) alters neural SOM
- Brain stimulation over dmPFC simultaneously alters behavioral SOM
- This suggests a causal role of dmPFC in separating self and other representations

Authors

Marco K. Wittmann, Nadescha Trudel, Hailey A. Trier, Miriam C. Klein-Flügge, Alejandra Sel, Lennart Verhagen, Matthew F.S. Rushworth

Correspondence

marco.k.wittmann@gmail.com

In brief

Wittmann et al. find that, after disrupting activity in the dorsomedial prefrontal cortex, humans merge performance estimates concerning other people with performance estimates about themselves. This suggests that representations of self and others are inherently interlinked and that intact dmPFC activity is needed for separating the two.

Wittmann et al., 2021, Neuron *109*, 2353–2361
July 21, 2021 © 2021 The Author(s). Published by Elsevier Inc.
https://doi.org/10.1016/j.neuron.2021.05.027

CellPress

Neuron

CellPress
OPEN ACCESS

Article

Causal manipulation of self-other mergence in the dorsomedial prefrontal cortex

Marco K. Wittmann,[1,4,5,*] Nadescha Trudel,[1,4] Hailey A. Trier,[1] Miriam C. Klein-Flügge,[1] Alejandra Sel,[1,2] Lennart Verhagen,[1,3] and Matthew F.S. Rushworth[1]

[1]Wellcome Centre for Integrative Neuroimaging (WIN), Department of Experimental Psychology, Tinsley Building, University of Oxford, Mansfield Road, Oxford OX1 3TA, UK

[2]Centre for Brain Science, Department of Psychology, University of Essex, Wivenhoe Park, Colchester CO4 3SQ, UK

[3]Donders Institute for Brain, Cognition, and Behaviour, Radboud University, Nijmegen, the Netherlands

[4]These authors contributed equally

[5]Lead Contact

*Correspondence: marco.k.wittmann@gmail.com

https://doi.org/10.1016/j.neuron.2021.05.027

SUMMARY

To navigate social environments, people must simultaneously hold representations about their own and others' abilities. During self-other mergence, people estimate others' abilities not only on the basis of the others' past performance, but the estimates are also influenced by their own performance. For example, if we perform well, we overestimate the abilities of those with whom we are co-operating and underestimate competitors. Self-other mergence is associated with specific activity patterns in the dorsomedial prefrontal cortex (dmPFC). Using a combination of non-invasive brain stimulation, functional magnetic resonance imaging, and computational modeling, we show that dmPFC neurostimulation silences these neural signatures of self-other mergence in relation to estimation of others' abilities. In consequence, self-other mergence behavior increases, and our assessments of our own performance are projected increasingly onto other people. This suggests an inherent tendency to form interdependent social representations and a causal role of the dmPFC in separating self and other representations.

INTRODUCTION

Navigating social environments requires us to interact with other agents that behave similarly to ourselves. Other people's decisions influence how we decide ourselves (Garvert et al., 2015; Suzuki et al., 2016), and we keep estimates of our own and other people's performance levels and abilities (Boorman et al., 2013; Wittmann et al., 2016). Ability estimates—impressions of how well we and others perform certain actions—allow us to construct shared representations of ourselves and others when we pursue cooperative goals or compete against each other. As a result, representations of ourselves change dynamically with social context (Wittmann et al., 2018). Here we investigate the causal contribution of the dorsomedial prefrontal cortex (dmPFC) to maintaining an individualized sense of one's own and others' abilities. In particular, we show that estimates of our own abilities are merged increasingly with estimates of others' abilities after causal manipulation of the medial prefrontal cortex.

DmPFC neurons signal others' trial and error learning in action reversal tasks (Yoshida et al., 2011, 2012) and encode the relative fit of one's own to other's actions (Seo et al., 2014). In the human dmPFC, corresponding signals indicate co-occurrence of ability estimates for oneself and others and the specific ways in which they can become merged (Wittmann et al., 2016). During self-other mergence (SOM), people estimate their ability not just as a function of their own performance. Instead, they are also biased by the performance of other people, depending on their contextual relationship with them (cooperation or competition). DmPFC activity is directly linked to context-dependent SOM. This is especially apparent during estimation of other people's abilities, but it is also apparent, albeit in less direct ways, during estimation of self-ability. For these reasons, here we focus on SOM in relation to estimation of other people's abilities.

The existence of such signals in the dmPFC and their correlation with SOM can, however, be interpreted in two completely opposite ways. This is partly a consequence of potential ambiguities in interpretation of correlations between variation in behavior and variation in neural activity that occur across participants (Lebreton et al., 2019). As a consequence, such signals might be responsible for causing SOM or, conversely, for ensuring that it does not occur to an even greater degree. These two interpretations suggest fundamentally different perspectives regarding the nature of our sense of self and others during social interaction. According to the first perspective, our default predisposition, in the absence of dmPFC activity, is an atomic, isolated sense of each individual, and dmPFC activity gives rise to interactions in self-other representations. According to the second perspective, our default predisposition is for a contextually

This is an open access article under the CC BY license (http://creativecommons.org/licenses/by/4.0/).

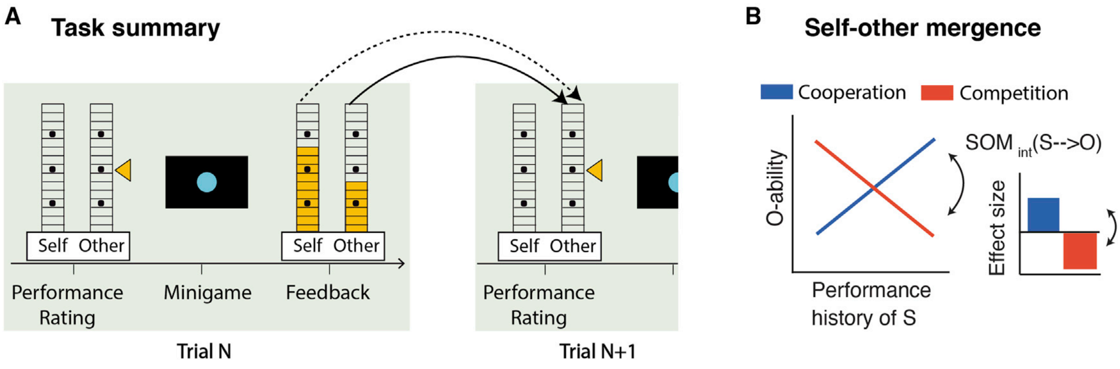

Figure 1. Social performance monitoring and SOM

(A) In each trial of the experiment, participants observed performance feedback for self and other relating to their respective performances in the minigame they had just played (higher yellow bars indicate better performance). Minigames, for example, comprised comparison of two time intervals between cues presented on the screen (see turquoise dots in the panel). Performance ratings made by participants at the start of each trial provided a behavioral readout of S-ability and O-ability estimation (in the example displayed here, the participant manipulates the yellow tick to predict the other player's performance). This allowed us to test how S- and O-ability estimates were based on recent performance feedback for the appropriate player (appropriate ability estimation; a solid arrow illustrates this for O-ability) and the inappropriate player (self-other mergence [SOM], dotted arrow; in this example, O-ability estimates are based on S-performance). Importantly, trials had a cooperative or competitive context, which is critical for SOM. Note that the task comprised two other players (STAR Methods); only one is shown here for illustration.

(B) Conceptual and mathematical formalization of SOM in the case of $SOM_{int}(S \rightarrow O)$ ($SOM_{int}(O \rightarrow S)$ operates analogously). During $SOM_{int}(S \rightarrow O)$, the estimation of O-ability is not just based on O-performance alone but also on S-performance. If S performed well recently, then O's performance is overestimated during cooperation but underestimated during competition. This means that the other player is estimated as worse or better in tandem with the current performance level of S. Blue and red slopes illustrate these linear relationships. These positive and negative slopes of the line plot are captured via our regression analysis approach by positive and negative effect sizes (bar graph); the bars summarize the strength and direction of *relationships* between the observed performances (S- and O-performance) and the resulting ability ratings (S- and O-ability estimates), allowing statistical testing of SOM as the difference between cooperative and competitive context. For the latter, we use the interaction of S-performance with social context. In this way, we quantify the absolute influence of S-performance on S-ability estimates while accounting for the inverted signs of the effects (positive in cooperation, negative in competition).

See also Figures S1–S3.

embedded and integrated sense of self and other, and separation is only effected by dmPFC activity. Arbitration between these two accounts can be achieved by determining the causal effect of dmPFC disruption: does the dmPFC disrupt or augment SOM? More specifically, in our previous work (Wittmann et al., 2016), dmPFC activity was directly correlated with the degree to which participants merged knowledge of their own performance (self-performance or S-performance) into estimates of others' abilities (O-ability estimates or O-ability) in a context-dependent manner. According to the first perspective, if the dmPFC enables SOM to occur, then dmPFC disruption should *decrease* SOM behavior and make O-ability estimates more independent from one's own performance. Alternatively, according to the second perspective, the dmPFC may be critical for keeping estimates of self and other separate, and higher dmPFC signals might have reflected an increase in activity aimed at preventing SOM from occurring. In this case, disrupting dmPFC activity should *increase* SOM behavior and make estimates of O-ability more dependent on S-performance.

Here we use a combination of non-invasive brain stimulation, neuroimaging, and computational modeling to assess the effects of causal manipulation of dmPFC activity on SOM in the estimation of other's abilities. Causal methods in addition to correlational measurements of brain activity, such as those provided by functional magnetic resonance imaging (fMRI), are indispensable for understanding brain function and have recently transformed our understanding of non-social decision-related signals in other brain networks (Ballesta et al., 2020; Knudsen and Wallis, 2020). Nonetheless, their use in social neuroscience is very rare (Hill et al., 2017). Here we show that, related to the estimation of another person, causal manipulation of the dmPFC diminishes neural signatures of SOM in the dmPFC and, in turn, increases behavioral SOM effects. This suggests that self and other ability estimates are inherently interdependent and that the dmPFC serves to keep them separate to ensure a correctly calibrated sense of self.

RESULTS

Measurements of appropriate performance estimation and SOM

We recently developed an experimental paradigm that allows precise measurement of how people form ability estimates (Wittmann et al., 2016). In this situation, people exhibit SOM; their estimates of their own ability are influenced by the performance of others, and, vice versa, their estimates of others' abilities are influenced by their own performance.

In the paradigm, participants perform arbitrary "minigames" (short reaction-time-based perceptual tasks) in each trial, and explicit performance feedback over many trials enables them to learn about their own abilities and those of two others. Figure 1 shows a simplified timeline of the central features of the task. We used predetermined performance feedback schedules to carefully match performance feedback for self and others and to

CellPress
OPEN ACCESS

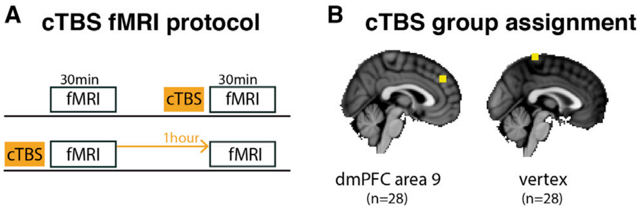

Figure 2. Neurostimulation protocol

(A) We collected fMRI data from each participant twice; once after cTBS stimulation and once in a control session. The order of cTBS and no stimulation blocks was counterbalanced across participants with a 1-h washout period between the sessions.

(B) One group (n = 28) received stimulation of the area of interest, dmPFC area 9, whereas a second group (n = 28) received stimulation of a non-active control region, the vertex. dmPFC stimulation coordinates were chosen based on our previous report of neural correlates of SOM in the dmPFC (Montreal Neurological Institute [MNI] target x/y/z coordinates in millimeters: 2/44/36). cTBS was compared with an order-balanced control condition as shown in (A).

keep them stable across participants. This ensured that performance learning for self and others was comparable across participants and, therefore, that individual differences in task behavior were interpretable. Subjects were told that the performance feedback reflected their objective performance mapped onto a 15-point performance scale and that the previously established mapping was the same for all players. Therefore, participants received explicit and independent performance feedback for all players. Using not only one but two minigames per session in pseudo-random trial order made it possible to have, on the one hand, slowly drifting performance shifts within a minigame (because abilities are thought to be relatively stable features) but, on the other hand, reduced sequential correlations across trials and ensured a full parametric range of performance feedback, making it possible to perform event-related fMRI analysis. Trial-wise decisions to engage and avoid cooperation/competition with a specified other player ensured that the social context was meaningful and that performance levels of all players were considered carefully by the participants (see STAR Methods and Figures S1–S3 for details regarding the minigames, engage/avoid decisions, and the other two players). Critically, performance ratings at the start of each trial provide a detailed readout of participants' estimates of their own ability (self-ability estimation: S-ability) and another player's ability (O-ability estimation). In general, participants perform this task correctly by using what we refer to as appropriate ability estimation—basing S-ability estimates on S-performance and basing O-ability estimates on O-performance. However, at the same time, ability estimates for self and others are intermixed (Figure 1A; Figures S1–S3). SOM is dependent on the social contexts in which trials occur, cooperation or competition, which require participants to entertain different relationships to the other player. During cooperation, high S-performance leads to overestimation of O-ability estimates, whereas in competition, high S-performance decreases estimates of O-ability estimates (Figure 1B). Corresponding SOM effects occur for S-ability estimation. SOM has been linked to activity in the medial prefrontal cortex. The strength of SOM effects correlates with fMRI signal strength in dmPFC area 9, suggesting that the dmPFC coordinates ability estimates for self and other (Wittmann et al., 2016).

Neurostimulation protocol

Here we used the same experimental paradigm to causally manipulate SOM by targeting its neural correlates in the dmPFC with 40-s continuous theta burst stimulation (cTBS) (Huang et al., 2005; Polanía et al., 2018) while recording the effect of this causal intervention on behavioral measurements of SOM and simultaneously acquired neural measurements of SOM. cTBS is an offline brain stimulation protocol that decreases cortical excitability of the targeted region for several minutes after application (Huang et al., 2005). cTBS has been used not only to modulate cortical excitability in motor areas but also in brain areas relevant for social cognition (Hill et al., 2017) and metacognition (Miyamoto et al., 2021). A group of participants underwent a shortened version of the experiment in the MRI scanner, preceded by cTBS (cTBS condition). After a temporal delay to guarantee washout of cTBS effects, participants underwent a second session without preceding cTBS (no-cTBS condition; Figure 2A). Session order was counterbalanced across participants (half of the participants performed the no-cTBS session first). Manipulating superficial features of the minigames (Figure S3) allowed us to present the exact same sequence of performance feedback in both sessions. This ensured that any observed SOM differences could only be attributable to the cTBS intervention. In addition to the within-participant control implemented by the two-session design, we used a between-participant control: in parallel, we recruited a same-sized second group of participants who underwent the same procedure, with the only difference being that cTBS was applied over the vertex instead of the dmPFC, a non-active control region where no SOM correlates have been identified previously (Wittmann et al., 2016; Figure 2B). Overall, these procedures resulted in 112 sessions of neural and behavioral data and allowed us to control for application of cTBS per se by testing whether the SOM difference in dmPFC-cTBS and no-cTBS sessions was bigger or smaller than the difference between vertex-cTBS and no-cTBS sessions. We applied the analogous analysis pipeline as previously to the behavioral and neural data (Wittmann et al., 2016) comprising a combination of reinforcement learning modeling (to capture S-performance and O-performance), behavioral regression analysis (to measure SOM effect sizes), and parametric fMRI analysis (to identify neural correlates). An important feature of the task design ensured that SOM is the consequence of the other agent's performance and not simply due to variation in payoff received in minigames (see Experimental task in the STAR Methods).

SOM in the baseline data

There was clear evidence of SOM in our experiment when examining data from only no-cTBS sessions collapsed over both groups. As expected, despite small differences in the relative strengths of effects, S-ability was influenced more positively by O-performance in cooperation than in competition ($SOM_{int}(O \rightarrow S)$), where "int" denotes the interaction term; $t(55) = 2.162$, $p = 0.035$; Figure 3A). At the same time, O-ability was similarly influenced more positively by S-performance in cooperation than competition ($SOM_{int}(S \rightarrow O)$); $t(55) = 3.233$, $p = 0.002$;

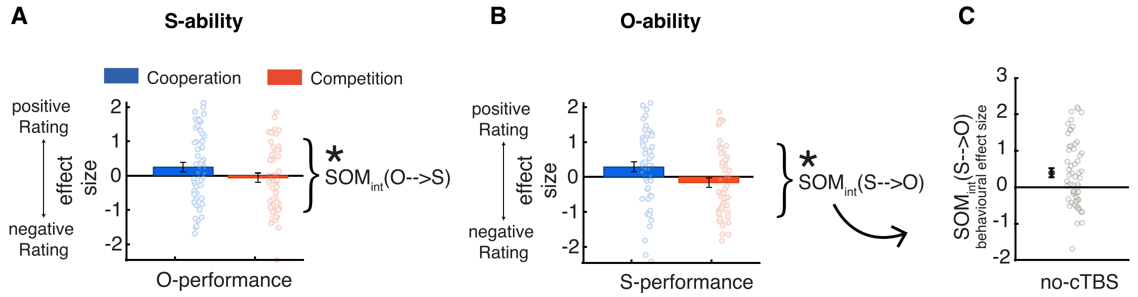

Figure 3. Behavioral SOM in the baseline data

(A and B) SOM is present in the no-cTBS data of the current experiment. Controlling for appropriate ability estimation (Figure 1A), S-ability estimates increase with increasing O-performance during cooperation relative to competition when S-ability estimates decrease with increasing O-performance ($SOM_{int}(O \rightarrow S)$, where "int" denotes the interaction term; see Figure 1B for an intuitive explanation of the effect sizes). The same difference is apparent when participants estimate O-ability with more positive influence of S-performance in cooperation compared with competition ($SOM_{int}(S \rightarrow O)$).

(C) Each SOM effect can be expressed by a single number indexing the difference in effect sizes between contexts (shown here: S-performance × context in O-ability rating; $SOM_{int}(S \rightarrow O)$). Positive $SOM_{int}(S \rightarrow O)$ indicates that O-ability is influenced more positively by S-performance in cooperation compared with competition. The strength of the behavioral $SOM_{int}(S \rightarrow O)$ effect was, in our previous report, directly related to a neural $SOM_{int}(S \rightarrow O)$ signal in the dmPFC (Figure S4).

Data are represented as mean ± SEM, and * denotes $p < 0.05$. See also Figure S4.

Figure 3B). Therefore, our experiment provided sensitive measurements of behavioral SOM. As previously (Wittmann et al., 2016), we define SOM by reference to the difference between cooperative and competitive trials; i.e., as the interaction of S-performance (O-performance) with the cooperative/competitive context (Figure 3C; Figure S4). This takes into account the fact that the influence of S-performance on O-ability estimates is positive in cooperation but negative in competition; during cooperation, we overestimate others' abilities at times when we perform very well ourselves, but in competition, we underestimate others' abilities when we are performing very well currently. Note that SOM is a decision-related rather than learning-related effect (SOM effects reflect the current social context of competition or cooperation between the S and O as opposed to the previous social context that prevailed when S and O last interacted). Moreover, our experimental design was constructed carefully to ensure that trial outcomes and winnings were decorrelated from S- and O-abilities (see Experimental task in the STAR Methods).

The full general linear model (GLM) from which the statistical results in Figures 3A and 3B are derived is shown in Figure 4 and is identical to one used previously by Wittmann et al. (2016). The filled bars in Figure 4 indicate the relevant SOM_{int} effects. The analysis shown in Figure 4 controls for appropriate ability estimation (the effect of S-performance on S-ability estimates and the effect of O-performance on O-ability estimates). Moreover, by including the "context" variable (coded as 1 for cooperation and −1 for competition), it also controls for the fact that people estimate O-ability per se as more positive in cooperation than in competition ($t(55) = 16.652$, $p < 0.001$), but the same is not the case for S-ability ($t(55) = -0.494$, $p = 0.624$). This may reflect an optimism bias in the estimation of O-ability—estimating O-ability in a way that would lead participants to believe they will maximize cooperative and competitive success. Importantly, by showing these effects in the same GLM as the SOM_{int} effects, we demonstrate that the SOM_{int} effects persist even after controlling for appropriate ability estimation and possible optimism biases. This result highlights the specificity and independence of the SOM effect: ability estimation for each player is affected by the performance of the other player in a manner that is dependent on the social context.

Disruption of the dmPFC silences neural signatures of SOM

In a previous study (Wittmann et al., 2016), one of the two SOM effects, $SOM_{int}(S \rightarrow O)$, was directly related to neural activity in the dmPFC (in contrast, dmPFC activity was not directly correlated with $SOM_{int}(O \rightarrow S)$ but instead with other measures of influence of O-performance on S-ability). Just as the effect of S-performance on O-ability changed depending on social context, the dmPFC carried an S-performance signal that was significantly different in cooperation compared with competition (S-performance × context). We refer to this signal as the neural $SOM_{int}(S \rightarrow O)$ signal. Strikingly, in our past report, behavioral and neural SOM effects correlated across participants, suggesting that neural activity in the dmPFC is critical for coordinating estimates of self and other abilities (Wittmann et al., 2016). Here we examine whether disturbing neural $SOM_{int}(S \rightarrow O)$ representations in the dmPFC is possible with cTBS and whether any such changes in neural computations cause measurable changes in behavioral $SOM_{int}(S \rightarrow O)$. Specifically, the dmPFC might coordinate self/other representations in one of two ways. If dmPFC constructs shared representations between self and other (for instance, to guide joint actions; Tomasello et al., 2005), then dmPFC disruption should *decrease* behaviorally observed $SOM_{int}(S \rightarrow O)$, making estimates of O-ability more independent from S-performance. Alternatively, the dmPFC may be critical for keeping estimates of self and other separate, in a manner akin to the way that the lateral orbitofrontal cortex maintains separate representations of choices so that outcomes are attributed correctly to the choices that caused them and not to other choices made close in time (Seo et al., 2014; Walton et al., 2010). In this case, disturbing dmPFC activity should *increase* behaviorally observed $SOM_{int}(S \rightarrow O)$, making estimates of O-ability less distinguishable from S-performance.

CellPress
OPEN ACCESS

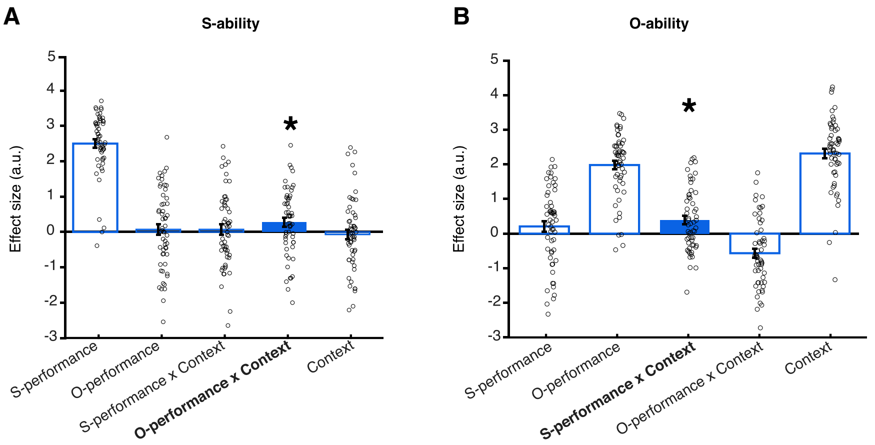

Figure 4. Full behavioral GLMs used for the baseline data

(A and B) The full GLM used to estimate S-ability (A) and O-ability (B) applied to the no-cTBS data (collapsed over the no-cTBS sessions in the dmPFC and vertex groups). These are the GLMs used to derive the statistical significance for the effects shown in Figure 3. Regressors included S-performance and O-performance, the trial's social context (cooperate/compete, coded as 1/−1), as well as the relevant interaction terms (see Rating GLM2 for S - with interaction by social context and Rating GLM2 for O - with interaction by social context for details). Filled blue bars highlight SOM interaction (SOM_{int}) effects. SOM regressors were calculated as the interaction of context and the relevant performance history (S-performance × context for O-ability regression [A] and O-performance × context for S-ability regression [B], respectively). Figures 3A and 3B show these effects in GLMs that separate cooperation and competition trials (see Rating-GLM1 for S - binned by social context [cooperate/compete] and Rating-GLM1 for O - binned by social context [cooperate/compete] for details). Significance testing, however, as done previously (Wittmann et al., 2016), was conducted on the SOM effects, indicated by the filled bars.

Data are represented as mean ± SEM, and * denotes $p < 0.05$.

We directly investigated neural $SOM_{int}(S \rightarrow O)$ effects in a sphere (16-mm radius) around our dmPFC stimulation coordinates ($z > 3.1$, $p = 0.05$ family-wise error [FWE] corrected; Table 1; related effects are shown in Figure S5) as in a recently reported cTBS-fMRI study (Hill et al., 2017). Because the vertex group provided a control for application of cTBS per se, we compared the cTBS effects in the dmPFC group (cTBS − no-cTBS) directly with the vertex group (cTBS − no-cTBS). Strikingly, we found a significant change in neural $SOM_{int}(S \rightarrow O)$ when applying cTBS to the dmPFC (Figure 5A; $SOM_{int}(S \rightarrow O)$ in the dmPFC/vertex × cTBS/no-cTBS interaction), indicating a stronger difference in $SOM_{int}(S \rightarrow O)$ effect sizes in the dmPFC compared with the vertex group. Closer inspection of the causal effect of cTBS on neural $SOM_{int}(S \rightarrow O)$ revealed that they were mostly driven by a strong reduction of S-performance signals in competitive trials in the dmPFC compared with the vertex group (Figure 5B; S-performance during competition in the dmPFC/vertex × cTBS/no-cTBS interaction). This effect was even strong enough to survive whole-brain correction ($z > 3.1$, $p = 0.05$ FWE). A significant reduction in S-performance effect size during competition was also present when examining the dmPFC group in isolation (Figure 5C; dmPFC, cTBS/no-cTBS). This indicates that neural $SOM_{int}(S \rightarrow O)$ was reduced after cTBS to the dmPFC (Figure 5D; see Figure S6 for corresponding vertex data). One reason why the cTBS effect might have been particularly pronounced during competition is that S-performance was especially strongly represented under this condition in the dmPFC during no-cTBS. The dmPFC might be particularly important in scenarios where representations of self and other must be kept separate, and this is particularly the case during competition. Therefore, cTBS-induced disruption of dmPFC activity weakened neural $SOM_{int}(S \rightarrow O)$.

For transparency, Figure 6 shows unthresholded brain-wide effects for the contrasts presented in Figure 5. No additional significance tests were performed on these whole-brain maps. In addition to the dmPFC region of interest (ROI), additional ROIs relevant for social cognition are highlighted: perigenual anterior cingulate cortex (pgACC), subgenual ACC (sgACC), and posterior temporoparietal junction (pTPJ). These regions are included as visual aids for locating relevant activations. The pTPJ is, apart from the dmPFC, perhaps the main ROI in social cognition research, and activity in this area often co-occurs with signals encoded in the dmPFC. We have taken a pTPJ mask from a recent study identifying subregions within the TPJ according to resting-state functional connectivity (Mars et al., 2012). The pgACC and sgACC were recently identified as carrying social signals (Lockwood and Wittmann, 2018; Lockwood et al., 2016; Will et al., 2017), and these regions are of particular relevance to the current study; social signals were identified in the pgACC in our previous work (Wittmann et al., 2016). We therefore took an ROI centered on the S-performance effect from that previous study (Wittmann et al., 2016). The sgACC is of particular interest in light of a recent combined cTBS-fMRI study that finds that the effect of cTBS on social computations in the TPJ spreads to the dmPFC but also to the sgACC (Hill et al., 2017). This suggests that similar network effects might be observable in our study. Effect sizes in Figures 6B and 6C are also high in the pTPJ. The pTPJ and dmPFC are part of a wider social cognition network, making it plausible that some information is transferred or shared between both areas.

Disruption of the dmPFC increases behavioral SOM

Finally we assessed whether the effect of cTBS on neural $SOM_{int}(S \rightarrow O)$ was sufficient to alter behavioral $SOM_{int}(S \rightarrow O)$. We went back to our behavioral regression, analyzing O-ability ratings using the index of behavioral $SOM_{int}(S \rightarrow O)$ illustrated in Figures 3C and 4. We performed the same comparison as with the fMRI data, comparing the difference in behavioral effect sizes for cTBS and no-cTBS conditions between the dmPFC and vertex groups. Causal manipulation of behavioral SOM by dmPFC neurostimulation was again evident when we examined behavior. There was a significant interaction between group (dmPFC/vertex) and stimulation (cTBS/no-cTBS) (2-way mixed effects ANOVA, $F(1,54) = 6.681$, $p = 0.012$; Figure 7).

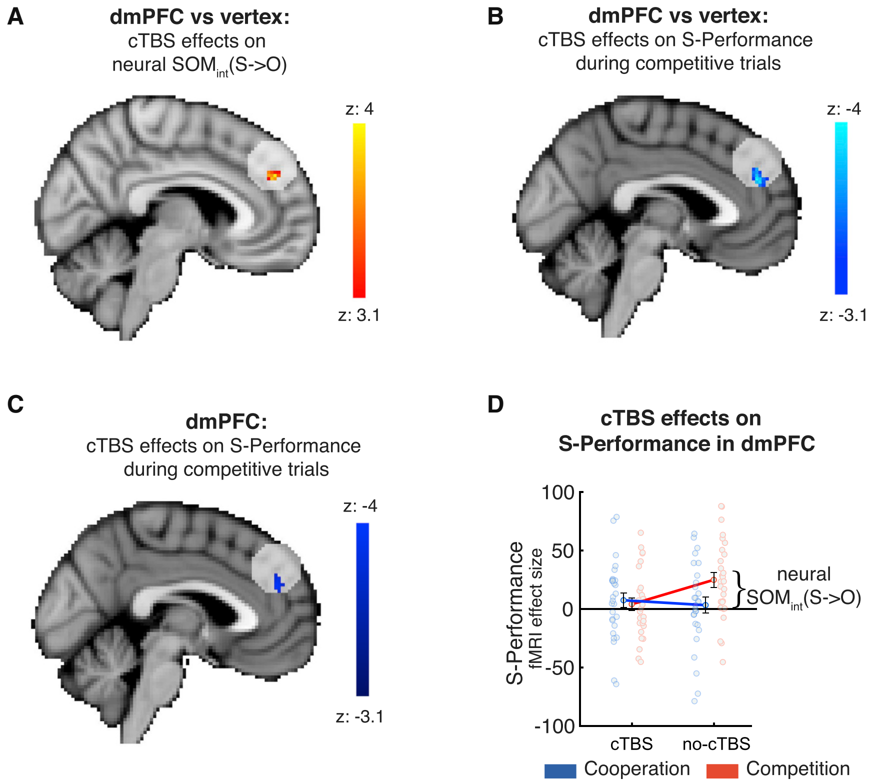

Figure 5. DmPFC-cTBS alters neural and behavioral SOM_{int}(S→O)

(A–C) Neural changes of SOM_{int}(S→O) in a sphere around the stimulation coordinates (pre-threshold masked, z > 3.1, p = 0.05 FWE).

(A) There is a stronger difference in SOM_{int}(S→O) between cTBS and no-cTBS sessions in the dmPFC group compared with the control group (SOM_{int}(S→O): dmPFC [cTBS – no-cTBS] > vertex [cTBS – no-cTBS]); the yellow cluster indicates a more positive difference in SOM_{int}(O→S) effect sizes in the dmPFC group compared with the vertex group.

(B) Closer inspection reveals that this effect is mainly driven by competitive trials in which S-performance is represented more weakly after cTBS stimulation of the dmPFC (left: dmPFC [cTBS – no-cTBS] > vertex [cTBS – no-cTBS]; the blue cluster indicates a more negative difference of S-performance effect sizes in the dmPFC group compared with the vertex group during competition). The effect is negatively signed; i.e., S-performance is represented relatively more weakly during dmPFC cTBS.

(C) Considering only the dmPFC group, S-performance in competition is decreased significantly in the cTBS compared with the no-cTBS condition (dmPFC [cTBS – no-cTBS]; again, the sign of this effect is negative).

(D) To illustrate the directionality of the effects shown in previous panels, (C) illustrates the neural effect sizes from the cluster shown in (B), right side, for S-performance in the dmPFC group. Application of cTBS reduces the difference between S-performance effect sizes in cooperation and competition; i.e., neural SOM_{int}(S→O) is reduced after cTBS application (blue/red indicate cooperation/competition trials).

Data are represented as mean ± SEM. See also Figures S5 and S6.

SOM_{int}(S→O) was increased significantly in the dmPFC group when applying cTBS (paired t test, $t(27) = 2.578$, $p = 0.016$), but this was not the case in the vertex control group. Within the dmPFC group, there was no significant correlation of neural (contrast of parameter estimates [COPE] images extracted from the cluster in Figure 5B) and behavioral SOMint(S→O) (Pearson $r = -0.205$, $p = 0.295$). This suggests that dmPFC cTBS causally increases SOM_{int}(S→O) in behavior and, hence, decreases the degree to which assessments of others' abilities are independent of knowledge about one's own performance. This effect was behaviorally specific (Figure S7). In contrast, mean neural and behavioral signatures of the complementary effect, SOM_{int}(O→S), were not affected by cTBS over the dmPFC. We did find, however, that the dmPFC induced correlated changes in SOM_{int}(O→S) behavior and SOM_{int}(O→S) neural activity; people who showed the greatest reduction in SOMint(O→S) neural activity with dmPFC cTBS showed the greatest increase in SOM_{int}(O→S) behavior (Figure S8).

Table 1. MNI peak coordinates of activation clusters

Contrast	Peak coordinates x/y/z (mm)	z value
S-performance × context (neural SOM_{int}(S→O)), dmPFC area 9 (cTBS – no-cTBS) > vertex (cTBS – no-cTBS) (GLM1)	6/46/30	3.94
S-performance during competitive trials, dmPFC area 9 (cTBS – no-cTBS) > vertex (cTBS – no-cTBS) (GLM2)	−4/46/24	−4.27
S-performance during competitive trials, dmPFC area 9 (cTBS – no-cTBS) (GLM2)	0/44/26	−3.91

All contrasts were calculated in spherical ROIs (16-mm radius) centered on the cTBS stimulation site in the dmPFC (MNI x/y/z coordinates in mm: 2/44/36) prior to thresholding.

DISCUSSION

Using non-invasive brain stimulation, fMRI, and computational modeling, we show that disruption of dmPFC causally affects neural signatures of SOM and the expression of SOM in behavior. The dmPFC is a key brain region for social-cognitive function (Ruff and Fehr, 2014; Saxe, 2006; Schurz et al., 2014) and encodes conspecifics' actions and outcomes in macaque monkeys (Noritake et al., 2018; Yoshida et al., 2012) and in humans (Piva et al., 2019; Sul et al., 2015; Suzuki et al., 2012).

CellPress
OPEN ACCESS

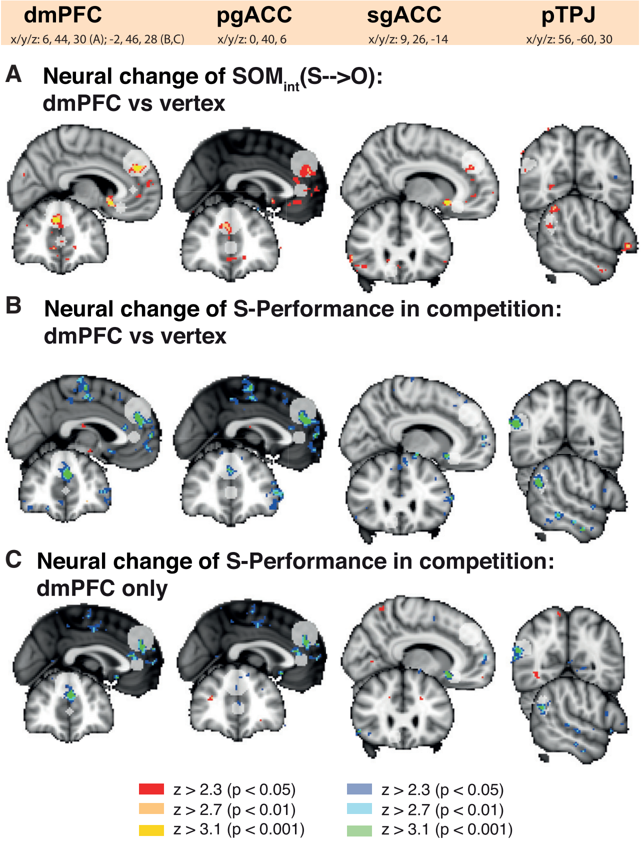

Figure 6. Brain-wide subthreshold effects related to SOM

Brain-wide effects are shown for the contrasts presented in Figure 5. Colors present uncorrected z-maps thresholded at $z > 3.1$, $z > 2.7$, and $z > 2.3$ (red colors indicate positive effects, and blue colors indicate negative effects). For transparency, further to a dmPFC ROI, additional ROIs relevant for social cognition are highlighted: perigenual ACC (pgACC) (MNI coordinates, x/y/z: 0/40/6), subgenual ACC (sgACC) (MNI coordinates, x/y/z: 9/26/−14; taken from Hill et al., 2017), and posterior temporoparietal junction (pTPJ) (anatomical mask from Mars et al., 2012).

(A) There is a stronger difference in $SOM_{int}(S \rightarrow O)$ between cTBS and no-cTBS sessions in the dmPFC group compared with the control group ($SOM_{int}(S \rightarrow O)$: dmPFC [cTBS – no-cTBS] > vertex [cTBS – no-cTBS]). The effect is positively signed.

(B) The $SOM_{int}(S \rightarrow O)$ effect in the dmPFC is driven mainly by the competitive trials where S-performance is represented more weakly after cTBS stimulation of the dmPFC (left, S-performance during competition: dmPFC [cTBS – no-cTBS] > vertex [cTBS – no-cTBS]). The effect is negatively signed.

(C) Considering only the dmPFC group, S-performance in competition is decreased significantly in the cTBS compared with the no-cTBS condition (S-performance during competition: dmPFC [cTBS – no-cTBS]). As in (B), again, the sign of this effect is negative.

See also Figures S5 and S6.

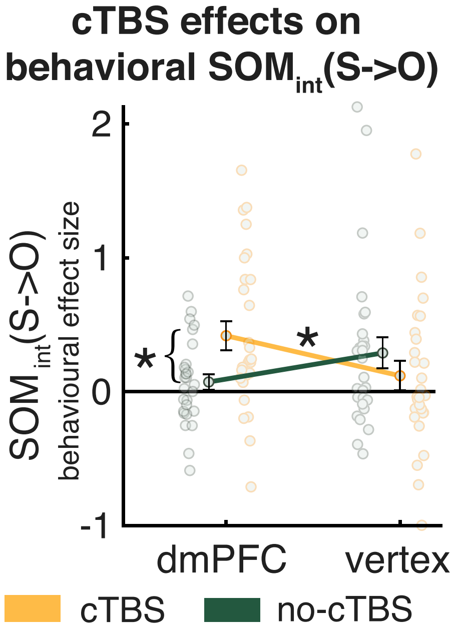

Figure 7. dmPFC cTBS alters behavioral $SOM_{int}(S \rightarrow O)$

The neural changes in $SOM_{int}(S \rightarrow O)$ (Figure 4D) translate into behavioral changes in $SOM_{int}(S \rightarrow O)$. $SOM_{int}(S \rightarrow O)$ is increased when applying cTBS to the dmPFC compared with no-cTBS, and this effect is stronger than the effect observed under the vertex control condition (yellow/green indicate cTBS/no-cTBS groups). Data are represented as mean ± SEM, and * denotes $p < 0.05$. See also Figures S7 and S8.

Correlative signatures of one's own and others' performance in the dmPFC predict the degree to which SOM occurs and people confuse their own ability with the ability of others and vice versa (Wittmann et al., 2016). We show that disruption of the dmPFC weakened these neural signatures of SOM. As a consequence, the degree to which people exhibit behavioral SOM increased; the ability estimated for the other player was influenced more positively by S-performance in cooperation compared with competition. We show the existence of these effects while controlling for a variety of possible confound effects, such as appropriate ability estimation (the influence of O-performance on O-ability estimation and the influence of S-performance on S-ability estimation) and optimism biases afforded by the social context.

Analogously, in the non-social decision-making domain, the brain holds representations not just of individual choices and their values but also representations that reflect their value in aggregate. For example, a region on the boundary between the lateral orbitofrontal cortex and ventrolateral prefrontal cortex, 47/12o, appears to be critical for forming and maintaining representations of specific choice values based on appropriate links between choices and rewards (Howard and Kahnt, 2018; Rudebeck et al., 2017; Walton et al., 2010). In contrast, other signals in the anterior insula and amygdala, respectively, reflect how good the environment is in general—the global reward state (Wittmann et al., 2020)—and less precise estimates of choice value (Chau et al., 2015; Jocham et al., 2016; Klein-Flügge et al., 2019). These brain regions are very different from those usually linked to social cognition, such as the TPJ, the gyrus of the anterior cingulate cortex, or the dmPFC (Apps et al., 2016; Dal Monte et al., 2020; Lockwood et al., 2018; Ruff and Fehr, 2014). However, it may be that different brain networks exhibit a relative specialization for social or non-social information based on their connectivity but that some of the computations performed in them are qualitatively similar (Hunt and Hayden, 2017).

Disruption of the dmPFC decreases neural signatures of SOM and increases the degree to which people merge estimates of their own performance with estimates of other's performance. This suggests a default tendency for representations of the self and of others to interact as a function of social context. Such a predisposition may have served a group-living species well in many situations where an individual's strength is a function of their allies' and opponents' strengths (De Dreu et al., 2016; Schülke et al., 2010; Wittmann et al., 2018) but may produce surprising and even problematic effects when social comparison operates in a wider sphere, as in contemporary society (Allport, 1924; Festinger, 1954; Toma et al., 2010). The neural representation of S-performance in the dmPFC serves to keep estimates of one's own and others' abilities separate and is essential for a correctly calibrated sense of self.

STAR★METHODS

Detailed methods are provided in the online version of this paper and include the following:

- KEY RESOURCES TABLE
- RESOURCE AVAILABILITY
  - Lead contact
  - Materials availability
  - Data and code availability
- EXPERIMENTAL MODEL AND SUBJECT DETAILS
  - Participants
- METHOD DETAILS
  - Pre-experimental procedure
  - cTBS stimulation sites
  - TMS protocols
  - fMRI experiment
  - Experimental task
  - Details on design, schedule, ability ratings, and performance feedback
- QUANTIFICATION AND STATISTICAL ANALYSIS
  - Reinforcement learning modeling
  - Details on reinforcement learning model architecture
  - Behavioral data analysis
  - Behavioral GLM analysis
  - Imaging Data Acquisition and Preprocessing
  - fMRI whole brain analysis
  - First-level fMRI analyses
  - Region of interest (ROI) analyses

SUPPLEMENTAL INFORMATION

Supplemental information can be found online at https://doi.org/10.1016/j.neuron.2021.05.027.

ACKNOWLEDGMENTS

We thank Katharina Angerer, Alberto Lazari, and Olof van der Werf for help with data collection. The study was funded by Wellcome Trust grant WT100973AIA (to M.F.S.R.), a Sir Henry Wellcome fellowship (103184/Z/13/Z to M.C.K-F.), a Bial Foundation grant (44/16 to A.S.), and Wellcome Trust Centre grant 203139/Z/16/Z. Work was carried out in the OHBA TMS lab funded by John Fell/Wellcome Trust Institutional Strategic Support Fund.

AUTHORS CONTRIBUTIONS

M.K.W., N.T., and M.R.S.R. designed the experiments. M.K.W., N.T., and H.A.T. collected the data. L.V. and M.C.K.-F. supervised the TMS setup. M.C.K.-F., L.V., and A.S. supported data collection. M.K.W. carried out data analyses. M.K.W., N.T., M.C.K.-F., L.V. and M.F.S.R. interpreted the results. M.K.W., N.T., and M.F.S.R. drafted the manuscript, and all authors commented on the manuscript.

DECLARATION OF INTERESTS

The authors declare no competing interests.

Received: November 8, 2020
Revised: March 30, 2021
Accepted: May 19, 2021
Published: June 24, 2021

REFERENCES

Allport, F.H. (1924). The group fallacy in relation to social science. J. Abnorm. Soc. Psychol. *19*, 60–73.

Apps, M.A., Rushworth, M.F., and Chang, S.W. (2016). The Anterior Cingulate Gyrus and Social Cognition: Tracking the Motivation of Others. Neuron *90*, 692–707.

Ballesta, S., Shi, W., Conen, K.E., and Padoa-Schioppa, C. (2020). Values encoded in orbitofrontal cortex are causally related to economic choices. Nature *588*, 450–453.

Boorman, E.D., O'Doherty, J.P., Adolphs, R., and Rangel, A. (2013). The behavioral and neural mechanisms underlying the tracking of expertise. Neuron *80*, 1558–1571.

Chau, B.K., Sallet, J., Papageorgiou, G.K., Noonan, M.P., Bell, A.H., Walton, M.E., and Rushworth, M.F. (2015). Contrasting Roles for Orbitofrontal Cortex and Amygdala in Credit Assignment and Learning in Macaques. Neuron *87*, 1106–1118.

Dal Monte, O., Chu, C.C.J., Fagan, N.A., and Chang, S.W.C. (2020). Specialized medial prefrontal-amygdala coordination in other-regarding decision preference. Nat. Neurosci. *23*, 565–574.

De Dreu, C.K., Gross, J., Méder, Z., Giffin, M., Prochazkova, E., Krikeb, J., and Columbus, S. (2016). In-group defense, out-group aggression, and coordination failures in intergroup conflict. Proc. Natl. Acad. Sci. USA *113*, 10524–10529.

Deichmann, R., Gottfried, J.A., Hutton, C., and Turner, R. (2003). Optimized EPI for fMRI studies of the orbitofrontal cortex. Neuroimage *19*, 430–441.

Eklund, A., Nichols, T.E., and Knutsson, H. (2016). Cluster failure: Why fMRI inferences for spatial extent have inflated false-positive rates. Proc. Natl. Acad. Sci. USA *113*, 7900–7905.

Festinger, L. (1954). A Theory of Social Comparison Processes. Hum. Relat. *7*, 117–140.

Garvert, M.M., Moutoussis, M., Kurth-Nelson, Z., Behrens, T.E., and Dolan, R.J. (2015). Learning-induced plasticity in medial prefrontal cortex predicts preference malleability. Neuron *85*, 418–428.

Hill, C.A., Suzuki, S., Polania, R., Moisa, M., O'Doherty, J.P., and Ruff, C.C. (2017). A causal account of the brain network computations underlying strategic social behavior. Nat. Neurosci. *20*, 1142–1149.

Howard, J.D., and Kahnt, T. (2018). Identity prediction errors in the human midbrain update reward-identity expectations in the orbitofrontal cortex. Nat. Commun. *9*, 1611.

Huang, Y.Z., Edwards, M.J., Rounis, E., Bhatia, K.P., and Rothwell, J.C. (2005). Theta burst stimulation of the human motor cortex. Neuron *45*, 201–206.

Huang, Y.-Z., Chen, R.-S., Rothwell, J.C., and Wen, H.-Y. (2007). The after-effect of human theta burst stimulation is NMDA receptor dependent. Clin. Neurophysiol. *118*, 1028–1032.

CellPress
OPEN ACCESS

Hunt, L.T., and Hayden, B.Y. (2017). A distributed, hierarchical and recurrent framework for reward-based choice. Nat. Rev. Neurosci. *18*, 172–182.

Jenkinson, M., Beckmann, C.F., Behrens, T.E.J., Woolrich, M.W., and Smith, S.M. (2012). FSL. Neuroimage *62*, 782–790.

Jocham, G., Brodersen, K.H., Constantinescu, A.O., Kahn, M.C., Ianni, A.M., Walton, M.E., Rushworth, M.F., and Behrens, T.E. (2016). Reward-Guided Learning with and without Causal Attribution. Neuron *90*, 177–190.

Klein-Flügge, M.C., Wittmann, M.K., Shpektor, A., Jensen, D.E.A., and Rushworth, M.F.S. (2019). Multiple associative structures created by reinforcement and incidental statistical learning mechanisms. Nat. Commun. *10*, 4835.

Knudsen, E.B., and Wallis, J.D. (2020). Closed-Loop Theta Stimulation in the Orbitofrontal Cortex Prevents Reward-Based Learning. Neuron *106*, 537–547.e4.

Lebreton, M., Bavard, S., Daunizeau, J., and Palminteri, S. (2019). Assessing inter-individual differences with task-related functional neuroimaging. Nat. Hum. Behav. *3*, 897–905.

Lockwood, P.L., and Wittmann, M.K. (2018). Ventral anterior cingulate cortex and social decision-making. Neurosci. Biobehav. Rev. *92*, 187–191.

Lockwood, P.L., Apps, M.A., Valton, V., Viding, E., and Roiser, J.P. (2016). Neurocomputational mechanisms of prosocial learning and links to empathy. Proc. Natl. Acad. Sci. USA *113*, 9763–9768.

Lockwood, P.L., Wittmann, M.K., Apps, M.A.J., Klein-Flügge, M.C., Crockett, M.J., Humphreys, G.W., and Rushworth, M.F.S. (2018). Neural mechanisms for learning self and other ownership. Nat. Commun. *9*, 4747.

Mars, R.B., Sallet, J., Schüffelgen, U., Jbabdi, S., Toni, I., and Rushworth, M.F. (2012). Connectivity-based subdivisions of the human right "temporoparietal junction area": evidence for different areas participating in different cortical networks. Cereb. Cortex *22*, 1894–1903.

Mazziotta, J., Toga, A., Evans, A., Fox, P., Lancaster, J., Zilles, K., Woods, R., Paus, T., Simpson, G., Pike, B., et al. (2001). A probabilistic atlas and reference system for the human brain: International Consortium for Brain Mapping (ICBM). Philos. Trans. R. Soc. Lond. B Biol. Sci. *356*, 1293–1322.

Miyamoto, K., Trudel, N., Kamermans, K., Lim, M.C., Lazari, A., Verhagen, L., Wittmann, M.K., and Rushworth, M.F.S. (2021). Identification and disruption of a neural mechanism for accumulating prospective metacognitive information prior to decision-making. Neuron *109*, 1396–1408.e7.

Noritake, A., Ninomiya, T., and Isoda, M. (2018). Social reward monitoring and valuation in the macaque brain. Nat. Neurosci. *21*, 1452–1462.

O'Shea, J., Johansen-Berg, H., Trief, D., Göbel, S., and Rushworth, M.F. (2007). Functionally specific reorganization in human premotor cortex. Neuron *54*, 479–490.

Piva, M., Velnoskey, K., Jia, R., Nair, A., Levy, I., and Chang, S.W.C. (2019). The dorsomedial prefrontal cortex computes task-invariant relative subjective value for self and other. eLife *8*, e44939.

Polanía, R., Nitsche, M.A., and Ruff, C.C. (2018). Studying and modifying brain function with non-invasive brain stimulation. Nat. Neurosci. *21*, 174–187.

Rossini, P.M., Barker, A.T., Berardelli, A., Caramia, M.D., Caruso, G., Cracco, R.Q., Dimitrijević, M.R., Hallett, M., Katayama, Y., Lücking, C.H., et al. (1994). Non-invasive electrical and magnetic stimulation of the brain, spinal cord and roots: basic principles and procedures for routine clinical application. Report of an IFCN committee. Electroencephalogr. Clin. Neurophysiol. *91*, 79–92.

Rudebeck, P.H., Saunders, R.C., Lundgren, D.A., and Murray, E.A. (2017). Specialized Representations of Value in the Orbital and Ventrolateral Prefrontal Cortex: Desirability versus Availability of Outcomes. Neuron *95*, 1208–1220.e5.

Ruff, C.C., and Fehr, E. (2014). The neurobiology of rewards and values in social decision making. Nat. Rev. Neurosci. *15*, 549–562.

Saxe, R. (2006). Uniquely human social cognition. Curr. Opin. Neurobiol. *16*, 235–239.

Schülke, O., Bhagavatula, J., Vigilant, L., and Ostner, J. (2010). Social bonds enhance reproductive success in male macaques. Curr. Biol. *20*, 2207–2210.

Schurz, M., Radua, J., Aichhorn, M., Richlan, F., and Perner, J. (2014). Fractionating theory of mind: a meta-analysis of functional brain imaging studies. Neurosci. Biobehav. Rev. *42*, 9–34.

Seo, H., Cai, X., Donahue, C.H., and Lee, D. (2014). Neural correlates of strategic reasoning during competitive games. Science *346*, 340–343.

Sul, S., Tobler, P.N., Hein, G., Leiberg, S., Jung, D., Fehr, E., and Kim, H. (2015). Spatial gradient in value representation along the medial prefrontal cortex reflects individual differences in prosociality. Proc. Natl. Acad. Sci. USA *112*, 7851–7856.

Suzuki, S., Harasawa, N., Ueno, K., Gardner, J.L., Ichinohe, N., Haruno, M., Cheng, K., and Nakahara, H. (2012). Learning to simulate others' decisions. Neuron *74*, 1125–1137.

Suzuki, S., Jensen, E.L., Bossaerts, P., and O'Doherty, J.P. (2016). Behavioral contagion during learning about another agent's risk-preferences acts on the neural representation of decision-risk. Proc. Natl. Acad. Sci. USA *113*, 3755–3760.

Toma, C., Yzerbyt, V., and Corneille, O. (2010). Anticipated cooperation vs. competition moderates interpersonal projection. J. Exp. Soc. Psychol. *46*, 375–381.

Tomasello, M., Carpenter, M., Call, J., Behne, T., and Moll, H. (2005). Understanding and sharing intentions: the origins of cultural cognition. Behav. Brain Sci. *28*, 675–691, discussion 691–735.

Trudel, N., Scholl, J., Klein-Flügge, M.C., Fouragnan, E., Tankelevitch, L., Wittmann, M.K., and Rushworth, M.F.S. (2021). Polarity of uncertainty representation during exploration and exploitation in ventromedial prefrontal cortex. Nat. Hum. Behav. *5*, 83–98.

Walton, M.E.M., Behrens, T.E.J.T., Buckley, M.J., Rudebeck, P.H., and Rushworth, M.F.S. (2010). Separable learning systems in the macaque brain and the role of orbitofrontal cortex in contingent learning. Neuron *65*, 927–939.

Will, G.J., Rutledge, R.B., Moutoussis, M., and Dolan, R.J. (2017). Neural and computational processes underlying dynamic changes in self-esteem. eLife *6*, e28098.

Wittmann, M.K., Kolling, N., Faber, N.S., Scholl, J., Nelissen, N., and Rushworth, M.F. (2016). Self-Other Mergence in the Frontal Cortex during Cooperation and Competition. Neuron *91*, 482–493.

Wittmann, M.K., Lockwood, P.L., and Rushworth, M.F.S. (2018). Neural Mechanisms of Social Cognition in Primates. Annu. Rev. Neurosci. *41*, 99–118.

Wittmann, M.K., Fouragnan, E., Folloni, D., Klein-Flügge, M.C., Chau, B.K.H., Khamassi, M., and Rushworth, M.F.S. (2020). Global reward state affects learning and activity in raphe nucleus and anterior insula in monkeys. Nat. Commun. *11*, 3771.

Yoshida, K., Saito, N., Iriki, A., and Isoda, M. (2011). Representation of others' action by neurons in monkey medial frontal cortex. Curr. Biol. *21*, 249–253.

Yoshida, K., Saito, N., Iriki, A., and Isoda, M. (2012). Social error monitoring in macaque frontal cortex. Nat. Neurosci. *15*, 1307–1312.

STAR★METHODS

KEY RESOURCES TABLE

REAGENT or RESOURCE	SOURCE	IDENTIFIER
Software and algorithms		
Jasp version 0.11.1	Jasp	RRID:SCR_015823
Presentation	Neurobehavioral systems	RRID:SCR_002521
MATLAB R2018a	MathWorks	RRID:SCR_001622
FSL	FMRIB, Oxford	RRID:SCR_002823
Brainsight	Rogue Research	RRID:SCR_009539
Spike2 Software	Cambridge Electronic Design Limited	RRID:SCR_000903
Behavioral data and analysis code	https://osf.io	https://accounts.osf.io/login?service=https://osf.io/h7jf8/
Others		
Magstim Rapid2 stimulator (TMS)	Magstim	https://www.magstim.com
D440 Isolated EMG amplifier	Digitimer	https://www.digitimer.com/
Hum Bug 50/60 Hz Noise Eliminator	Quest Scientific	https://www.digitimer.com/
CED power1401	Cambridge Electronic Design Limited	RRID:SCR_017282

RESOURCE AVAILABILITY

Lead contact

Further information and requests for resources and reagents should be directed to and will be fulfilled by the Lead Contact, Marco Wittmann (marco.k.wittmann@gmail.com).

Materials availability

This study did not generate new unique reagents.

Data and code availability

We have deposited all choice raw data in an OSF repository. Behavioral results in this paper are derived from these data alone.This repository also comprises the full MATLAB behavioral analysis pipeline including reinforcement learning model, regression analyses and plotting scripts. A README inside the repository explains the details of its use. The access code is: https://accounts.osf.io/login?service=https://osf.io/h7jf8/

EXPERIMENTAL MODEL AND SUBJECT DETAILS

Participants

65 participants participated in the fMRI experiment. Nine of those participants were excluded from the data analysis due to premature cessation of the experimental session (n = 2; no data were available for two participants who terminated the experiment after the *pre-experimental procedure* described below), technical difficulties with the task program or button box (n = 4), inability to follow the standardized experimental structure (n = 2) or report of the belief that the experiment involves deception (n = 1). The final sample contained 56 participants (age range 18-39 years, 26 female). Two of these 56 participants aborted a scanning session after the majority of the session was completed but prior to its full completion; their data was included in all analyses. Of these 56 participants, 28 were assigned to the dorsomedial prefrontal cortex (dmPFC) group and 28 were assigned to the vertex group. The two groups underwent identical experimental procedures that only differed in the stimulation site where transcranial magnetic stimulation (TMS) was applied. Our sample size is in line with a recent cTBS-fMRI study examining the causal effects of disrupting another node of the social brain network, the temporoparietal junction (Hill et al., 2017).

Subjects received £70 for participating in the fMRI experiment and two preceding behavioral sessions (see below). In addition, they received extra earnings which were allocated according to their task performance (mean = £13.96; std = £2.85; range: £6.23 - £18.18). The ethics committee of the University of Oxford approved the study and all participants provided informed consent (MSD-IDREC-C2-2015-017, MSD-IDREC-C1-2013-133).

METHOD DETAILS

Pre-experimental procedure

Before participating in the fMRI experiment, participants took part in two preparatory sessions on separate days. First, they took part in a ''taster session'' (~1.5h), which served to explain TMS as well as the experimental task to the participants, to perform face-to-face TMS safety screenings, and to measure participants' active motor thresholds. In addition, we also applied a short, 10 s version of the TMS used in the experiment, continuous theta burst stimulation (cTBS) (Huang et al., 2005), at a lower standardized intensity (25% of the machine output) over the approximate location of dmPFC. This was well below the duration and intensity required to yield neural effects and was meant to familiarize participants with the procedure that would be used on the final day in the fMRI experiment. On a subsequent day, participants were invited to a structural magnetic resonance image (MRI) brain scan (30 minutes overall), which was a prerequisite to perform neuronavigated cTBS on the final day during the fMRI experiment.

cTBS stimulation sites

During the fMRI experiment, we stimulated two separate groups of participants in two different target stimulation sites using cTBS. In one of the groups, we aimed to disrupt neural activity in dmPFC where we have previously identified neural correlates of self-other-mergence (SOM) (Wittmann et al., 2016). We used the relevant peak coordinates of effects found in our previous study in Brodmann area 9 (O-performance effects in Figure 3Aii/Table 1 of our previous report (Wittmann et al., 2016); Montreal Neurological institute (Mazziotta et al., 2001) (MNI) x/y/z coordinates in mm: 2/44/36). The stimulation site for the other group, the vertex, served as a non-active control condition and was defined as the intersection of the central sulci from both cortical hemishpheres (Hill et al., 2017) (MNI /x/y/z coordinates in mm: 0/-34/72). The fact that at this location in the brain the distance between brain tissue and skull is very large as well a lack of relevant neural activation found in this location in the previous study (Wittmann et al., 2016) meant that the vertex was an appropriate control stimulation site. Both stimulation sites were defined in standard MNI space and warped to participant specific structural images using FMRIB Software Library's (FSL) (Jenkinson et al., 2012) non-linear transformations (FNIRT). All participants were blinded as to the site where they were stimulated with cTBS and experimental procedures, including the neuronavigation setup, were identical for all participants. Experimenters were necessarily aware of the stimulation site in order to be able to apply cTBS.

TMS protocols

All TMS stimulation was applied with a Magstim-rapid-2 stimulator (MagStim, Whitland, Carmarthenshire, UK) connected to a 70mm figure-8 coil. We used TMS on two occasions: on the first day during the 'taster session' to measure participants' active motor threshold (Rossini et al., 1994) and during the fMRI experiment on the final day to apply neuronavigated cTBS (Huang et al., 2005). We used the same TMS coil on both occasions.

During the taster session on the first day, we assessed participants' active motor threshold for the left motor cortex 'hotspot', which is the scalp location where TMS evoked the largest MEP amplitude. The active motor threshold was defined as the minimum stimulation intensity sufficient to produce a motor-evoked potential (MEP) in the contralateral small hand muscle, i.e., right first dorsal interosseous (FDI), in at least 50% of trials, when the participants exerted a constant pressure between the index finger and the thumb (20% of maximum force) (Rossini et al., 1994). Electromyographic (EMG) activity in right FDI was recorded with bipolar surface Ag-AgCl electrode montages. Responses were bandpass filtered between 10 and 1000 Hz, with additional 50 Hz notch filtering, sampled at 5000 Hz, and recorded using a CED 1902 amplifier, a CEDmicro1401 Mk.II A/D converter, and PC running Spike2 (Cambridge Electronic Design).

On the day of the fMRI experiment, we performed a standard neuronavigated cTBS protocol (Huang et al., 2005) immediately prior to one of two fMRI sessions that participants performed on the day. The stimulation site was projected onto the high-resolution, T1-weighted MRI brain scan of each participant using frameless stereotactic neuronavigation (Brainsight; Rogue Research). Inion, nasion, right ear and left ear were used for registration of the structural image. The stimulation protocol comprised 600 pulses in bursts of three pulses at 50Hz that are applied every 200 ms following a procedure first described by Huang et al. (2005). When applied over the motor cortex projecting to muscles from the contralateral hand, cTBS reduces motor-evoked potentials recorded from these hand muscles. This reduction in motor output generated from the motor cortex is likely to be caused by reduced efficacy of synaptic transmission lasting approximately half an hour (Huang et al., 2007; Polanía et al., 2018). A TMS coil was held in place tangentially to the skull by an experimenter during stimulation. The total stimulation duration was 40 s. We obtained the cTBS intensity by taking 80% of the output of the TMS machine at each subject's active motor threshold. So, for example, if a participant's motor threshold lies at 45% of the TMS machine's maximal output, the stimulation intensity was 80%*45% = 36% of the machine's maximal output. The use of such a low subthreshold intensity (80% active motor threshold) had the advantage of ensuring decreased spread of stimulation away from the targeted site. For dmPFC stimulation, due to the proximity to facial nerves, we tested participants with a maximal cTBS intensity of 45% percent output of the maximal TMS machine (mean = 35.5%, std = 4.3%, range = 27% - 44%). For vertex, we used a stimulation intensity of maximal 51% machine output (mean = 42.2%, std = 6.9%, range = 29% - 51%), which was the highest available output intensity for the TMS machine. Participants for which a stimulation intensity higher than these thresholds were determined after the initial taster session did not go on to participate in the fMRI experiment. Participants rested for 40 s after the end of the stimulation to avoid washout as in previous studies (O'Shea et al., 2007). Afterward, participants went from the

stimulation room to the directly adjacent scanning room and started the fMRI session immediately. The scan sequence started approximately 5 minutes after the end of the cTBS application (including the 40 s rest). Participants took approximately half an hour to perform the experimental session in the MRI scanner. Any unnecessary movements were held to a minimum in this process.

fMRI experiment

On the day of the fMRI experiment, participants were again screened for TMS and MRI safety and received a reminder of the task instructions. They performed two experimental sessions in the fMRI scanner (Figure 2). Both sessions lasted approximately 30 minutes and were separated in time by at least one-hour. During a break between the two sessions, participants relaxed and filled in some questionnaires unrelated to the purpose of the TMS manipulation (~20 minutes). One of the sessions was preceded by the cTBS stimulation. No cTBS manipulation was performed preceding the other session. Importantly, the order of the two sessions was counterbalanced across participants. Prior to both fMRI sessions, participants performed a short 'starter-session' on a desktop computer. The experimental task during the starter-session was identical to the fMRI session and served to familiarize participants with the task before entering the scanner and to ensure that in-scanner behavior was maximally informative (see Experimental task section below). The starter-session took 5-7 minutes. One of the two starter-sessions was immediately followed by cTBS (neuronavigation and target localization had been performed before the starter-session). Participants also performed a starter-session before the other fMRI session, but they completed an unrelated questionnaire for 4 minutes between the starter-session and fMRI session to mirror the temporal delay imposed by the cTBS procedure. This means that, for participants that had cTBS in the first session, the mock questionnaire was completed in the second session, whereas for participants who had the cTBS in the second session, the mock questionnaire was completed in the first session. The length of the mock questionnaire matched approximately the duration of the cTBS application. The questionnaires were discarded after the experiment and no analyses were performed on them. In the end, participants were fully debriefed about the experiment.

Experimental task

We used an experimental paradigm established in our previous work (Wittmann et al., 2016) that we summarize in the following. Additional details are described in detail in the section below. Modifications of the paradigm only served to adapt it to the requirements of a paired cTBS-fMRI experiment and are detailed after the summary of the experimental task below in this section.

In the paradigm, participants learned about the approximate performance levels of themselves and two other players over trials. The only way to do this was to track explicit, parametric performance feedback for each player that was provided at the end of the trial. Prior to the performance feedback, participants performed one iteration of a so-called minigame, i.e., a short, reaction-time based task with a continuous performance scale. Participants were instructed to perform as well as possible in these minigames and believed the performance feedback that was subsequently presented reflected how well they themselves and the other players had performed in it. Note that the performance feedback only referred to the immediately preceding run of the minigame; it was not aggregate feedback over a longer time period. Approximate performance levels could only be learned by tracking performance feedback over several trials and using a recency-weighted average score to predict future performance. While participants believed that the performance feedback originated from their true performance in the minigame, it was in fact taken from a pre-determined performance schedule that was adapted from our previous study (Wittmann et al., 2016). However, exceptions from this pre-determined schedule existed and constituted cases of veridical performance feedback; this was an additional measure to assure that performance feedback was believable (see section below). Participants were told that the performance of the other player was pre-recorded on a different day.

Before the performance feedback was provided and the minigame performed, participants made an engage/avoid decision and subsequently rated the expected performance of themselves (S) and one of the two other players (*relevant other*, O). O was pseudorandomly determined by the experimenter. The engage/avoid decision was set in either a cooperative or competitive context (experimenter-determined, pseudo-random). It reflected either the decision to engage in or avoid a cooperative relationship with O on the current trial (as opposed to playing individually), or the decision to engage in or avoid a competitive relationship with O on the current trial (as opposed to playing individually). Importantly, trials either offered the cooperative decision or the competitive decision; there was no choice between cooperation and competition. This allowed us to study contextual effects on the ratings that participants made about their own and O's performance feedback on the current trial. The participant's goal was to predict the performance feedback that they themselves and O would receive at the end of the trial and the payoff scheme incentivized them to do this as accurately as possible. To do this, they had to carefully track the performance history of each player and the ratings provided a detailed readout of participants' current player-specific performance estimates. Importantly, the experimental design was carefully constructed to ensure that trial outcomes and winnings were decorrelated from both S and O abilities. For example, on trials on which S and O performances were high, the threshold which participants had to surpass to win a trial might also be high. This meant that performance estimates for both players were dissociable from general reward expectation. This should also minimize the impact of any reinforcement-related effects on ability estimations. More detailed description of the paradigm is given the section below. Instructions are explained in detail in Figure S1 and Figure S2 presents a detailed timeline of trial events.

We modified several aspects of the paradigm to adapt it to cTBS-fMRI. First, for both fMRI sessions, we used the identical performance schedule, i.e., the series of trials and the performance feedback given was exactly the same in both sessions. This allowed us to assess participant's neural mechanism of self and other performance learning twice on the same performance schedule with the

CellPress
OPEN ACCESS

only difference being the application of cTBS before one session and not the other. However, to avoid any learning effects from one session to the next one, we designed separate minigames for each fMRI session ('time minigames' in one session and 'color minigames' in the other; Figure S3). This, together with the instructions, created the impression that both sessions measured different aspects of cognitive performance. Participant were told they played with other players during each session. No participant realized that they performed the same schedule twice. The assignment of the time and color minigames to the first or second fMRI session was counterbalanced across participants and orthogonal to the application of cTBS. Second, we shortened the number of trials of the fMRI session to 88 trials per session for participants to be able to complete the session within a time frame over which cTBS effects could be assumed to last (Huang et al., 2005). We also slightly shortened the timings of some trial events to accommodate as many trials as possible in the 30 minutes window (see Figure S2 for timings). Third and finally, we introduced 'starter-sessions' to each fMRI session (see fMRI experiment section). These starter-sessions employed the same minigames as the respective fMRI session and their performance schedule naturally preceded the one administered in the fMRI session. However, as our paradigm requires learning over trials, the initial trials of each session cannot provide meaningful readouts of participants' performance estimates. Participants first need to form initial impressions about each players' performance levels before these can be assessed. As cTBS effects diminish quickly, the usage of starter-sessions allowed us to shift this behaviorally uninformative time period to a time before cTBS was applied. Starter-sessions preceded the cTBS application. They lasted for 16 trials with no engage/avoid decisions and no ratings performed during the initial four trials. Starter-sessions were similarly performed prior to the other fMRI session with an appropriate break mirroring the time delay cause by cTBS application (see fMRI experiment section). This enabled us to assess the effects of cTBS on self and other performance learning from the very first trial of the fMRI sessions onward.

Details on design, schedule, ability ratings, and performance feedback

In this section, we give some additional details on experimental task details that we also provided when we introduced the paradigm previously (Wittmann et al., 2016). On each trial, participants performed a minigame (short reaction time-based tasks) and received performance feedback for all three players involved. Participants were told that the minigames had been tested on a larger sample of participants and that performance feedback in the minigame reflected individual performance relative to that sample. In the phases before and after the minigames, three scales ranging from 1-15 points were shown with the initials of the three players below. Performance feedback was displayed on these scales in the feedback phase. The initials shown were adjusted to be appropriate for each individual participant. The initials created a social frame for the experiment without using explicitly social cues such as faces.

On each trial, participants also made an engage/avoid decision and rated the expected performance for themselves and a *relevant other* (O); except the first four trials of the starter-session (see Experimental task). The identity of O (whether it was the player shown to the right or to the left of the participant on the screen) was experimenter-determined and pseudo-random. Each trial took place either in a cooperative or in a competitive social context. In a cooperative trial the engage/avoid decision was between cooperating or refraining from cooperating, while on a competitive trial, the choice was between competing or refraining from competing. If participants took the "avoid" choice, then that meant that they simply either won or lost a small number of points (1.5 points) at the end of the trial. Win or loss occurred with the same probability and hence the "avoid" choice had an expected value of zero on average. Participants were informed about this. However, if participants took the "engage" choice in the cooperative context then they opted to ally themselves with O to see if together they could perform well enough for their average points to exceed a threshold level (which varied from trial to trial and was explicitly cued on the screen). If they did exceed the threshold after engaging, they gained reward points on that trial, but if they fell short of the threshold, they lost points. By contrast, if they took the "engage" decision in the competitive context then the other player became an opponent. The difference between the participant's and opponent's performances then had to exceed a threshold (again the threshold was variable). In both cooperation and competition, the reward points earned or lost were proportional to this difference to the threshold (i.e., a win, a loss or neither of the two). In summary, the social context was critical when decisions to engage were made. Reward outcomes for engage/avoid choices were determined by minigame performances of S, O, and a threshold that varied unpredictably from trial to trial:

$$EngagePayoff_{Competition} = (feedback_S - feedback_O) - threshold \tag{1a}$$

$$EngagePayoff_{Cooperation} = (feedback_S + feedback_O)/2 - threshold \tag{1b}$$

"Feedback" in Equations 1A and 1B refers to performance feedback observed at the end of the trial for S and O and "threshold" abbreviates the height of the cued threshold on the trial. While the likely performance feedback for S and O could be estimated from performance feedback on previous trials, the threshold varied unpredictably from trial to trial and was used to dissociate reward expectation from performance expectation and to make sure that participants did not make their engage/avoid decisions before the beginning of the current trial. Participants found the meaning of the thresholds intuitive when the task was being explained to them and their task behavior confirmed that they had understood the task. See Figure S1 for task instructions.

Participants then also provided an estimate of their ability on each trial by rating the expected performance for themselves (S) and the *relevant other* (O) for the upcoming trial of the minigame. The order of S and O ratings was randomized across trials. As explained above, although both of the two other players performed the minigame simultaneously, participants were only paired (to compete or

cooperate) with one of the other players (the *relevant other*, O). Therefore, only O, and not the third player, was relevant for a trial's engage/avoid decision. However, the identity of O switched between trials. On each trial, after the minigame, participants received performance feedback about themselves as well as about the performances of the other two players. See Figure S2 for a detailed trial timeline including timings of all trial events.

The goal of the participants in the experiment was to collect as many rewards (points) as possible, as these were translated into monetary rewards at the end of the experiment. Participants could achieve this by making correct engage/avoid decisions and by predicting performances accurately in the ratings. For all three players, including the participants themselves, performance feedback on every trial was predefined. In other words, the feedback about performance was independent from participants' actual performance in the minigames (see, however, "false start trials" for a case of veridical performance feedback in the *Feedback* part of this section below and Figure S2C). This was necessary to control and match performance feedback between participants as well as between participants and the two other players. Importantly, it allowed us to use the identical performance schedule for both fMRI sessions, i.e., the series of trials and the performance feedback given was exactly the same in both sessions. This was crucial to test the effects of cTBS on performance learning of self and other, as it was only the application of cTBS that differed while the experimental schedule was the same.

We designed two 'time minigames' and two 'color minigames' which were used for the first and second fMRI session. See Figure S3 for details on the minigames including timings. Across participants, we counterbalanced which one was shown in the first session and second session. Importantly, the order of the cTBS application (cTBS was either applied before the first or the second fMRI session) was randomized across participants and orthogonal to the minigame identity. In addition, other task features were counterbalanced across participants and orthogonal to those previous manipulations as well as to each other. These additional features include the mapping between minigame and associated performance feedback schedule for the participant, the association between the other players (left/right) and their performance feedback schedules, and the button mapping between left/right and engage/avoid choice.

The fMRI experimental schedule contained 88 minigame trials. The design was a 2[social context] x 2[partner] x 2[minigame] fully crossed design (11 trials per cell). This meant that a trial could be either cooperative or competitive [social context: cooperation or competition], the O could be either "player" 1 or 2 [O: Other1 or Other2] and, in each trial, participants played one of two minigames [minigame: game1 or game2]. The trial type order was pseudorandom and the same for all participants. The starter-session had 16 trials overall and comprised an approximately similar number of trials per trial type. The first four did not include an engage/avoid decision to allow participants to first learn about the approximate performance levels of each player.

In summary, trials comprised an engage/avoid decision, two ratings (for S and O), a minigame phase, and a feedback phase. Instructions are detailed in Figure S1, trial timelines including timings in Figure S2 and minigame features in Figure S3.

As already mentioned, participants provided S and O ability ratings. For each rating, initially, a tick indicated a value on the performance scale (*rating marker*) and participants indicated if expected performance (for S or O as appropriate) would surpass or fall below the rating marker (Figure S2B). A positive rating (i.e., performance is expected to be above the rating marker) was made with one button and a negative rating (i.e., performance is expected to be below the rating marker) was made with the other button. As performance feedback was always expressed in integers, the rating makers were always set between two integers (X.5-values) such that either of the two responses was always correct. The rating marker was updated from trial to trial based on the rating choices for the respective player using a staircasing procedure to increase sensitivity of the ratings. A positive rating resulted in an increase of the rating marker's value by one point in the next trial of the same minigame for the given player; a negative rating resulted in a decrease by one point. Participants received a small payoff for the accuracy of the ratings. To reduce incentives to perform badly on the minigames, negative ratings never yielded payoffs. For positive ratings, participants won or lost 0.25 points depending on whether the subsequent performance feedback received surpassed or fell below the rating marker. Note that the magnitude of the rating payoff was insignificant compared to the payoff for the engage/avoid decision.

Feedback was chunked together in three components which were presented in randomized order. The first component was the performance feedback for S and O, which was presented simultaneously with the information about the accuracy of the participants' ratings (Figure S2B). The second component was the payoff of the engage/avoid decision. For this, a cue indicating the trial's choice appeared on the right side of the screen (Figure S2A) together with circles representing coins that were won (yellow circles with a plus sign) or lost (red circles with a minus sign). At the same time, only for engage choices, the performance feedback average (cooperation trials) or performance feedback difference (competition trials) was displayed on the scales on the right side of the screen. The third component was the performance feedback of the other player that was not the O (irrelevant other). The initials of this player were displayed in a different color and the performance was irrelevant for any payoff. Note although the performance feedback for this player was irrelevant to the current trial, it would become relevant in the future when the currently irrelevant other would become the relevant other. The three feedback components appeared in random order to control for sequence effects.

Two types of trials deviated from the described structure. First, the first four trials of the starter-session (which took place outside of the scanner) were "starter trials" (two with one minigame, two with the other). Those trials were for participants to form initial ability estimates about the players. For this reason, in starter trials, there was no option to cooperate or compete and no ability ratings were made. Second, for trials where participants performed very badly in a minigame ("false starts") the feedback phase was adjusted. The performance thresholds for false start trials are discussed in Figure S3. In false start trials, participants received no performance feedback for themselves, but only for the other players (Figure S2C). The sole payoff for false start trials was a loss of three points

independent of participants' ratings and engage/avoid choice. Participants were instructed about this and asked to avoid producing false start trials. It was explained that extremely bad performances would be detected by the computer running the experiment and discarded as false starts to sort out performance slips that were, for instance, due to inattentiveness and did not reflect a player's "true" performance. This procedure was used to make the pre-determined feedback in other trials more believable as the feedback in false start trials was actually determined by true minigame performance. Note that participants were also told during the instructions that there would be a special type of false start trial if one of the other players performed very badly. However, this never happened in the experiment. Starter trials and the feedback phase of false start trials were excluded from fMRI analysis.

QUANTIFICATION AND STATISTICAL ANALYSIS

Reinforcement learning modeling

We used the exact same reinforcement learning (RL) model as in our previous work (Wittmann et al., 2016) (see Details on reinforcement learning model architecture section below). Again, as in our previous work, we submitted the computational variables from our fitted model to a general linear model (GLM) analysis predicting the rating data. The rating GLM was specifically designed to test for self-other-mergence (SOM; e.g., dependence on another player's performance when estimating one's own ability or dependence on one's performance when estimating another player's ability) as opposed to appropriate ability estimation (e.g., relying on one's own performance to estimate one's own ability, relying on the other player's performance when estimating that other player's ability). The RL model ensured that we could capture an index of the longer-term average performance levels observed for oneself and the other players – necessary prerequisites for the GLM analysis. The computational variables capturing these longer-term average performance levels were termed *S-performance* (for oneself) and *O-performance* (for the relevant other). Importantly the effects we tested for in the subsequent GLM were orthogonal to the RL model fitting because we tested for SOM effects, whereas the RL model only assumed appropriate ability estimation. To avoid bias in the model fitting, for each group (dmPFC and vertex), we fitted both fMRI sessions together for all participants, which resulted in a single set of free parameters per group (one set for the dmPFC and one for the vertex group). The modeling was implemented using MATLAB 2018a.

Details on reinforcement learning model architecture

For every participant, we fitted a standard reinforcement learning model to model the performance estimates assigned to the three players for each trial (Self, S; Other1, O1; Other2, O2). We used two minigame specific performance estimates per player (either for the two color minigames or the two time minigames). The performance estimates summarize the previous performance history of the players and are hence referred to as *performance*. They reflect the expected performance based on a recency-weighted average of past performance feedback. This resulted in six player and minigame specific *performance* estimates: $performance_{S\text{-}T1}$, $performance_{S\text{-}T2}$, $performance_{O1\text{-}T1}$, $performance_{O1\text{-}T2}$, $performance_{O2\text{-}T1}$, $performance_{O2\text{-}T2}$. T1/T2 denote the two session-specific minigames. On every trial t, the three *performance* estimates associated with the current minigame were updated using a prediction error (PE) based learning rule with a learning rate α as a free parameter:

$$Performance_{t+1} = performance_t + \alpha \times PE_t \tag{1}$$

(formula was applied separately for S, O1, O2, given T1 or T2)

The PE itself was calculated based on the specific *performance* estimate and performance feedback of the player in the current minigame as:

$$PE_t = feedback_t - performance_t \tag{2}$$

(formula was applied separately for S, O1, O2, given T1 or T2)

In false start trials, the *performance* estimate for S was not updated and remained unchanged until the next trial of the same minigame. No PEs for S were calculated for false start trials (the other players never displayed false start trials), but PEs were calculated for the other players. For the first trial of the fMRI session for each player for each minigame, the last performance feedback from the starter session in the respective minigame was taken as a starting value for *performance*.

In each trial in the fMRI session, participants made a decision about cooperating or competing (depending on the current context) with the relevant other (O) and in addition provided ratings of both S and O. Both engage/avoid decisions and ratings were modeled based on *performance* estimates for S and O, called *S-performance* and *O-performance*. *S-performance* is the *performance* estimate for Self associated with the minigame of the current trial ($performance_{S\text{-}T1}$ or $performance_{S\text{-}T2}$). Similarly, *O-performance* refers to $performance_{O1\text{-}T1}$, $performance_{O1\text{-}T2}$, $performance_{O2\text{-}T1}$ and $performance_{O2\text{-}T2}$, depending on which other player was currently selected as the O and which minigame took place. Therefore, *S-performance* and *O-performance* represented minigame and player specific performance expectations of the players involved in the current trial's engage/avoid decision. The same was the case for the PEs associated with S and O.

Participants' ratings of a player reflected expectations of whether they would perform either better or worse than a level indicated by a *rating marker* the position of which was adjusted from trial to trial using a staircase procedure explained in the above sections on experimental designs. Expectations expressed in the ratings that exceeded or fell below the rating marker were referred to as positive and negative ratings, respectively. To calculate the probability of a positive rating (p(positiveRating)), we used a softmax function with

an inverse temperature β. This was done separately for S and O using *S-performance* and *O-performance*, respectively as well as the player specific rating marker:

$$P(positive\ Rating) = \frac{\exp[\beta \times (performance - ratingmarker)]}{\exp[\beta \times (performance - ratingmarker)] + 1} \quad (3)$$

(this formula was applied separately for *S-performance* and *O-performance* given their respective rating markers)

Having calculated the probability of a positive rating on a given trial, the probability of the rating actually observed was derived, again, separately for S and O:

$$\mathrm{P(rating)} = \begin{cases} p(positive\ Rating) & if\ rated\ positively \\ 1 - p(positive\ Rating) & if\ rated\ negatively \end{cases} \quad (4)$$

(formula was applied separately for S and O)

Participants also received a small gain or loss at the end of a trial if they had made a positive rating and the expectation indicated by that rating had been accurate (rating bonus of 0.25 points). As explained in the Experimental Procedures, to ensure that there was no temptation to perform poorly in the task no rating bonus was awarded when a negative rating had been given. The expected value of a rating (EV_{rating}) was calculated as

$$EV_{rating} = \begin{cases} [p(positive\ Rating) - 0.5] \times 2 \times ratingbonus & if\ rated\ positively \\ 0\ if\ rated\ negatively \end{cases} \quad (5)$$

(formula was applied separately for S and O)

Note the formula was chosen such that the bounds of EV_{rating} for positive ratings are 0.25 and −0.25, which are the points that can be lost or won for positive ratings.

In addition to completing a rating for S and O on each trial, participants made a decision to engage in or avoid cooperating/competing. Given the objective payoff scheme of the task (Equations 1A and 1B from the Experimental Design section above), the expected value of engaging in cooperation/competition (EV_{engage}) was calculated in an analogous way:

$$Competition:\ EV_{engage} = S\text{–}performance - O\text{–}performance - threshold \quad (6a)$$

$$Cooperation:\ EV_{engage} = (S\text{–}performance + O\text{–}performance)/2 - threshold \quad (6b)$$

A decision to avoid cooperating/competing led to a gain of 1.5 points and a loss of 1.5 points with equal probability (see previous section on experimental design) and participants had been instructed that the expected value of the decisions to avoid cooperating/competing was zero:

$$EV_{EAD} = \begin{cases} EV_{engage} & if\ engage \\ 0 & if\ avoid \end{cases} \quad (7)$$

Therefore, EV_{engage} was used as decision variable for the engage/avoid decisions to calculate the probability of engaging in cooperation or competition:

$$P(engage) = \frac{\exp(\beta \times EV_{engage})}{\exp(\beta \times EV_{engage}) + \exp(\beta \times EV_{avoid})} \quad (8)$$

Note that EV_{avoid} is zero in Equation 8, as explained above. The probabilities of the actual choices made were derived from p(engage):

$$EV_{EAD} = \begin{cases} P(engage) & if\ engage \\ 1 - P(engage) & if\ avoid \end{cases} \quad (9)$$

The full reward expectation on each trial (EV_{chosen}) was defined as the sum of the expected values from both ratings and the expected value of the engage/avoid decision (Equations 5 and 7):

$$EV_{chosen} = EV_{S-Rating} + EV_{O-Rating} + EV_{EAD} \quad (10)$$

The reward prediction error (RPE) was calculated based on all reward outcomes of a trial including both rating reward outcomes and the engage/avoid decision reward outcome (see above Equation 2 for the calculation of player specific prediction errors):

$$RPE = Reward - EV_{chosen} \quad (11)$$

Overall, the free parameter set θ comprised two free parameters: the learning rate α and the inverse temperature β. We fitted these parameters by minimizing the negative log likelihood (nLL) over all trials N from both fMRI sessions from all participants of each group

together (dmPFC or vertex), resulting in one set of fitted free parameters per group. For the calculation of nLL, we treated ratings and engage/avoid decisions equally. So the decisions used to fit the model included equal proportions of ratings of S, ratings of the O and engage/avoid choices.

$$nLL = -\sum_{n=1}^{N} \log(p(decision_t|\theta)) \tag{12}$$

Behavioral data analysis

We analyzed behavioral data using MATLAB 2018a and Jasp version 0.11.1. We analyzed how participants estimated their own and O's ability by applying a logistic general linear model (GLM) regression to the rating data in which participants predicted the performance outcome for both players based on their observed history of past performance in the minigames. This allowed us to examine if and how TMS changed self-other-mergence. As described in the section Reinforcement learning modeling, we used a reinforcement learning model to capture an index of the longer-term average performance levels observed for oneself and the O. The two key variables from the model feeding into the GLM analyses were *S-performance* (recency-weighted performance estimate for self) and *O-performance* (recency-weighted performance estimate for relevant other). The critical effects of interest to measure self-other-mergence were the influence of *O-performance* on S-ability estimation and the influence of *S-performance* on O-ability estimation. The key effects of interest were the interactions of *S-performance* and *O-performance* with the *Context* variable (cooperation and competition, coded as 1/-1). Following this definition, $SOM_{int}(S \rightarrow O)$, for example, quantifies the context-dependent influence of S-performance influence on O-ability estimation. Importantly, by using the interaction term we quantify that influence in complementary ways in cooperation compared to competition, testing for a positive effect of *S-performance* on O-ability estimation in cooperation and in parallel for a negative effect of *S-performance* on O-ability in competition. The inclusion of *S-performance* in the S rating and *O-performance* in the O rating (the appropriate performances that should be used for ability estimates) only served as control variables in the GLMs. Note that all SOM GLM effects are orthogonal to the fitting done in the RL model, which only assumed appropriate ability estimation. We used the exact identical two GLMs we had used in our previous work with this paradigm (Wittmann et al., 2016). We describe them in detail in the section Behavioral GLM analysis below. After applying the logistic GLMs to each participant independently, based on the resulting beta weights for the SOM-related effects, we calculated a mixed effects ANOVA (group: dmPFC and vertex; condition: cTBS and no-cTBS) and also a paired t test for the dmPFC data alone (Figure 7). This constituted the most critical test of the cTBS effects on self-other-mergence. For demonstrating SOM effects in the baseline no-cTBS data (Figures 3 and 4), we used variance-weighted beta weights (MATLAB's stats.t object) as index of effect size to de-weight outlying data points as we have done previously (Trudel et al., 2021). The reason for the small trial number was to ensure that the experimental session would finish before cTBS effects subsided. The cTBS-induced changes we report (Figure 7), however, were calculated based on standard beta weights.

Behavioral GLM analysis

We used the exact identical two GLMs we had used in our previous work with this paradigm (Wittmann et al., 2016). As described in the section Reinforcement learning modeling in the STAR Methods, we used a reinforcement learning model to capture an index of the longer-term average performance levels observed for oneself and the O. The two critical variables from the model feeding into the GLM analyses were *S-performance* (recency-weighted performance estimate for self) and *O-performance* (recency-weighted performance estimate for relevant other). Note that all SOM GLM effects are orthogonal to the fitting done in the RL model, which only assumed appropriate ability estimation.

We used two rating GLMs that both comprised the same set of regressors of interest, but one of them binned trials into contexts of cooperate and compete trials (the two types of social context), whereas the other one took the interactions of *S-performance* and *O-performance* with *context.* Context was coded as 1 for cooperation and −1 for competition. As in our previous work (Wittmann et al., 2016), these interaction terms were the crucial measures for SOM. They are underlined in the formulas below: the influence of *O-performance* on the S rating and the influence of *S-performance* on the O rating. To calculate the interaction terms, we normalized the performance variable and multiplied it with the normalized context variable as we did before. Following the rationale of our previous work (Wittmann et al., 2016), we restricted both rating GLMs to trials in which participants had chosen to engage. Only in those trials, the social context of cooperation or competition is critical (rather than on "avoid" trials when participants simply took the default option of a random payment; see the Experimental task section and Figure S2). Note also that the use of *S-performance* in the S rating and *O-performance* in the O rating (the appropriate performances that should be used for ability estimates) only served as control variables in the GLMs. For all GLMs, all regressors (also interaction terms) were normalized (mean of 0 and standard deviation of 1).

First, the binned GLM, rating-GLM-1, was used for visualization only. It was applied separately to cooperate and compete trials:

Rating-GLM1 for S - binned by social context (cooperate/compete):

S-performance, *O-performance,* ratingmarker-S

Rating-GLM1 for O - binned by social context (cooperate/compete):

O-performance, *S-performance,* ratingmarker-O

Note that the "ratingmarker" refers to the position of the rating tick against which ability is estimated in the current trial (see the Experimental task section and Figure S2). As output, we used the variance-corrected beta weights (MATLAB's stats.t object) to account for the relatively low number of trials as we have done before (Trudel et al., 2021). Note that no effects in this GLM were tested for significance. The next GLM, rating-glm-2, used the same regressors, but instead of binning, it employed the interaction terms of performance with social context. These interaction effects were tested for significance of SOM_{int} ("int" denotes "interaction"). A positive interaction effect, for example for *S-performance* in the O rating, indicated that the effect of *S-performance* was stronger in cooperation than in competition. Again, the analyses were restricted to engage trials.

Rating GLM2 for S - with interaction by social context

S-performance, O-performance, S-performance x Context, O-performance x Context, Context, ratingmarker-S
(SOM_{int}(O→S) is underlined)

Rating GLM2 for O - with interaction by social context

S-performance, O-performance, S-performance x Context, O-performance x Context, Context, ratingmarker-O
(SOM_{int}(S→O) is underlined)

Imaging Data Acquisition and Preprocessing

Imaging data were acquired with a 3-Tesla Siemens MRI scanner by using a 32-channel head coil. T1 weighted structural images were collected with an echo time (TE) of 4.75msec, a repetition time (TR) of 3secs, an inversion time (TI) of 100msec, 1x1x1mm voxel size and a 256x176x224 grid. Functional images were collected by using a Deichmann echo-planar imaging (EPI) sequence with TE = 30msec, TR = 3sec, 3x3x3mm voxel size, 87° flip angle, 30° slice angle and z-shimming to avoid signal dropout in frontal areas such as medial orbitofrontal areas (Deichmann et al., 2003). Two fieldmap scans (sequence parameters: TE1, 5.19ms; TE2, 7.65ms; TR, 488 s; flip angle, 60 ; voxel size, 3.5 × 5.5 × 3.5 mm) of the B0 field were also acquired and used to assist distortion-correction.

FMRIB's Software Library (FSL) was used to analyze imaging data (Jenkinson et al., 2012). We pre-processed the data through fieldmap correction, and temporal (3 dB cut-off 100sec) and spatial filtering (Gaussian using full-width half maximum of 5mm) and using FSL's MCFLIRT to correct for motion. The functional scans were registered to standard MNI space using a two-step process: (1) registration of subjects' whole- brain EPI to T1 structural image was conducted using BBR with (nonlinear) fieldmap distortion-correction, and (2) registration of the subjects' T1 structural scan to 1 mm standard space was performed using an affine transformation followed by nonlinear registration. We used FSL's MELODIC to filter out noise components after visual inspection.

fMRI whole brain analysis

On the first level, we closely adapted the single GLM we had used in our previous work with this paradigm (Wittmann et al., 2016). It uses the identical set of regressors to model self and other ability estimation: *S-performance, O-performance,* and *context* as in our previous work. In addition, we constructed a second fMRI GLM that, instead of including the interaction terms (*S-performance x context*, *O-performance x context*), bins trials into cooperative and competitive trials to be able to analyze TMS effects separately in the two types of social contexts. These two GLMs operate therefore in a similarly complementary manner as the two behavioral GLMs (see section *Behavioral GLM analysis*) and are detailed in the First level fMRI analyses section below.

The results of the first level GLM were submitted to a fixed-effects second level analysis. For each participant, irrespective of dmPFC or vertex group, we calculated the difference in activation between the cTBS and the no-cTBS session. These difference maps were then submitted to a third-level analysis. We calculated two-sample unpaired t tests to compare whether the difference in neural activation change due to dmPFC stimulation was indeed stronger than the neural change in the vertex group. In addition, we also examined differences between the cTBS and no-cTBS sessions for the dmPFC group only using one-sample t tests. For statistical analysis of the third-level analysis, we used Flame 1+2 mixed effects analyses (Jenkinson et al., 2012). All results were FWE cluster corrected at $p < 0.05$ using a cluster-defining threshold of $z > 3.1$ ($p < 0.001$) (as recommended in Eklund et al., 2016) within an *a priori* defined search volume. Following previous studies examining cTBS induced neural changes (Hill et al., 2017), the search volume was a 3-dimensional sphere with 16mm radius. It was centered on our stimulation site in dmPFC, where we previously identified neural correlates of self-other mergence (MNI x/y/z coordinates in mm: 2/44/36).

First-level fMRI analyses

We used two fMRI GLMs for our whole brain analyses. Following the rationale of our behavioral analyses, the two GLMs employ the same set of regressors to model self and other ability estimation but differ in whether they model the social *context* (cooperation or competition) as an interaction effect or whether trials are binned into cooperative and competitive trials. The first GLM, the interaction GLM, was virtually identical to the one used in our previous work with this paradigm (Wittmann et al., 2016). Both GLMs employ *S-performance* and *O-performance* – indices of the longer-term average performance levels observed for oneself and the relevant other, O, derived from our reinforcement learning model (see Reinforcement learning model architecture section above and the Reinforcement learning modeling section). All parametric and binary regressors were normalized (mean of zero, standard deviation of one). The main phase of interest was the decision phase. However, we also modeled the feedback phase to account for variance associated with it and therefore also report feedback-related regressors for completeness.

As in our previous work (Wittmann et al., 2016), fMRI-GLM 1 time-locked the decision phase, as a constant regressor, to the onset of the engage/avoid decision onset. The duration was set to the response time for the engage/avoid decision (Figure S2Aii: phase 1,2 and 3). We added several parametric regressors to the decision phase:

- *S-performance*
- *O-performance*
- *S-performance x Context,*
- *O-performance x Context,*
- *Context* (binary regressor; cooperation 1, competition −1)
- *Threshold*

The two interaction terms were calculated as explained in the Behavioral GLM analysis section above. As in our previous paper (Wittmann et al., 2016), we calculated the *Threshold* regressor (reflecting the performance that had to be reached to satisfy the cooperative or competitive choice; see Equations 1A and 1B in the Experimental paradigm section above) over all trials by combining the threshold regressors from cooperate and compete conditions and normalizing them separately for each condition. The timing parameters for the parametric regressors were identical with the constant decision phase regressor, except for the *Threshold*. The threshold onset was delayed by one second, as the threshold was only revealed one second after engage/avoid decision onset and knowledge of the threshold was necessary to make an engage/avoid decision (see phase 2 in Figure S2Ai). The duration of the *Threshold* regressor was set to the time between its onset and the response button press in the engage/avoid decision

As in our previous report (Wittmann et al., 2016), we used two constant regressors with a duration of zero time-locked to the response of S and O rating to account for the rating events. In addition, we used parametric regressors accompanying these constant regressors accounting for the reward expectations associated with the ratings (EV_{rating} for S and O from Equation 5 in the Reinforcement learning model architecture section above for S rating and O rating, respectively).

The feedback phase was similarly modeled as a constant regressor and parametric modulators. Note that trial feedback was chunked in three components and presented in randomized order (see Experimental Paradigm section above and Figure S2).

I) S and O performance feedback and rating reward outcomes
II) Engage/avoid decision reward outcome
III) Irrelevant other performance feedback

Duration of the constant feedback regressor was 2.5 s, the time window in which the three feedback components initially appeared (phase 2 onset to phase 4 onset in Figure S2Aii). Parametric regressors were modeled as stick functions (i.e., duration of zero) time-locked to the appearance of the relevant feedback component (indicated in brackets in the following). They comprised:

- *S-performance* (I)
- *O-performance* (I)
- S-PE (Prediction error for Self) (I)
- O-PE (Prediction error for relevant other) (I)
- *Context* (I)
- O-PE x *Context* (I)
- Overall reward payoff from the trial (II)
- *Threshold* (II)
- Prediction error - irrelevant other (III)

Roman numerals in brackets after the regressors indicate to which feedback component a regressor was time-locked. Feedback phases from false start trials were not modeled.

In addition, the GLM contained three additional regressors of no interest. First, a regressor time-locked to all button presses, modeled as stick functions, to account for movement-related effects. Second, two regressors captured brain signals associated with each minigame, spanning the time period from minigame onset to response button press.

fMRI-GLM-2 modeled the same constant events as the previous GLM. It modeled the decision phase in exactly the same way, but separately for competitive and cooperative trials. It used the same parametric regressors of interest as above, but without the interaction terms:

- *S-performance*
- *O-performance*
- *Threshold*

The *Threshold* regressor was slightly time-delayed for the same reasons as above.

All remaining regressors modeled events of no interest. We report them for completeness. We again modeled constant regressors with a duration of zero time-locked to the response of S and O rating to account for the rating events, but separately for cooperative and competitive trials.

The feedback phases were also split for cooperative and competitive trials. We modeled the following parametric regressors time-locked to the different feedback phases (Roman numerals in brackets indicate the same phases as above):

- *S-performance* (I)
- *O-performance* (I)
- S-PE (Prediction error for Self) (I)
- O-PE (Prediction error for relevant other) (I)
- EV_{rating} for both ratings combined (I)
- reward prediction error for both EV_{rating} combined (I)
- S-pChange (I) [the absolute change in true minigame performance from one trial to the next one; see (Wittmann et al., 2016 for details]
- *Threshold* (II)

Region of interest (ROI) analyses

We illustrate the neural effects of interest by reading out FSL's COPE (contrast of parameter estimates) maps for both groups and both stimulation conditions. We extracted the S-performance effects in competitive and cooperative trials from fMRI GLM 2 (see *First level fMRI analyses* section; Figure 5D; Figure S5) in a mask that was derived from the contrast S-performance in competitive trials (Area 9 (cTBS – no-cTBS); see Figure 5D and Table 1) by thresholding it at z > 3.1.

Neuron, Volume 109

Supplemental information

Causal manipulation of self-other mergence in the dorsomedial prefrontal cortex

Marco K. Wittmann, Nadescha Trudel, Hailey A. Trier, Miriam C. Klein-Flügge, Alejandra Sel, Lennart Verhagen, and Matthew F.S. Rushworth

Supplementary figures

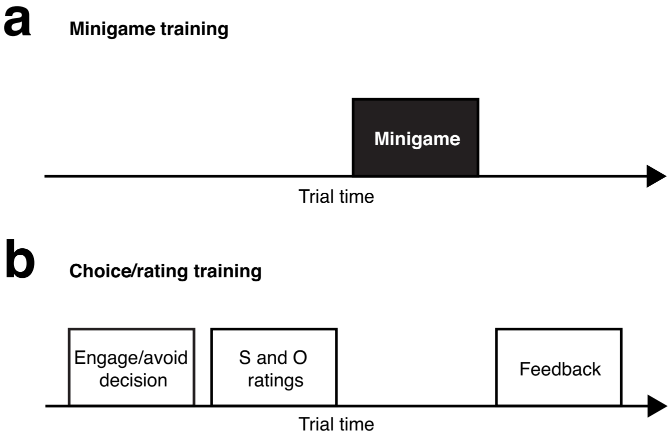

Figure S1. Instruction procedure. Related to Figure 1. The experimental instructions followed a precise schedule implemented according to our previous study (Wittmann et al., 2016). On the day of the 'taster session', when participants were introduced to TMS and their active motor thresholds were assessed, they also received full instructions for the experiment (~1 hour), which were briefly recapitulated on the day of the fMRI experiment. In the instructions, participants were told that they would play minigames during the experiment and their goal was to learn about their own and other players' performances and to make good decisions and ratings based on these pieces of information. It was emphasized that doing this would enable them to collect as many reward points as possible during the experiment and that they should aspire to do that. They were told that the performances of the other players that they would be paired with in the fMRI experiment was pre-recorded and they agreed that their own, fully anonymized performance data could be used for the same purpose in the future. Participants were instructed on the minigames, the ratings, and the engage/avoid decisions and performed example trials. The instructions were designed such that actual performance learning would only take place in the fMRI experiment to maximize learning effects in the scanner. (**a**) To still guarantee familiarity with the minigames, a written explanation of the two minigames was complemented with a short practice session, in which participants performed trials of each minigame. During those trials, the experimenter was present and made sure that participants understood the minigames. No explicit performance feedback was given on those trials to avoid performance learning. (**b**)To guarantee that participants understood the logic of the engage/avoid decision and the ratings, participants performed example trials that did not include minigames, but instead a placeholder screen. This allowed participants to adjust to the trial events and experience the reward outcomes of ratings and decisions. Importantly, the performance feedback on those trials followed no across-trial contingencies and consisted mostly of the highest or lowest performance feedback for the players. This was done to make these example trials very different from the trials experienced in the main experiment. Although participants could not learn anything during those trials and therefore could make no well-grounded decisions, they were asked to invent and verbalize reasons for their ratings and decisions so that the experimenter could make sure that they understood their logic (e.g. "This is a cooperate trial. I press the "engage" button, because although the threshold is high, I think we will perform very well. I rate myself and the other one positively, because I think we will both perform well..."). In sum, participants practiced all aspects of the experimental task, but in such a manner that they could not yet learn about their performance. Participants were told that their goal in the experiment was to collect as many points as possible and that points would be translated into monetary reward at the end of the experiment. It was emphasized that points could be earned by making good decisions and by providing accurate ratings of performance. Despite the substantial time needed for a thorough instruction, as in our previous study, most participants found the task intuitive and the behavioral data acquired in the experiment (see main text) confirmed that they understood the task.

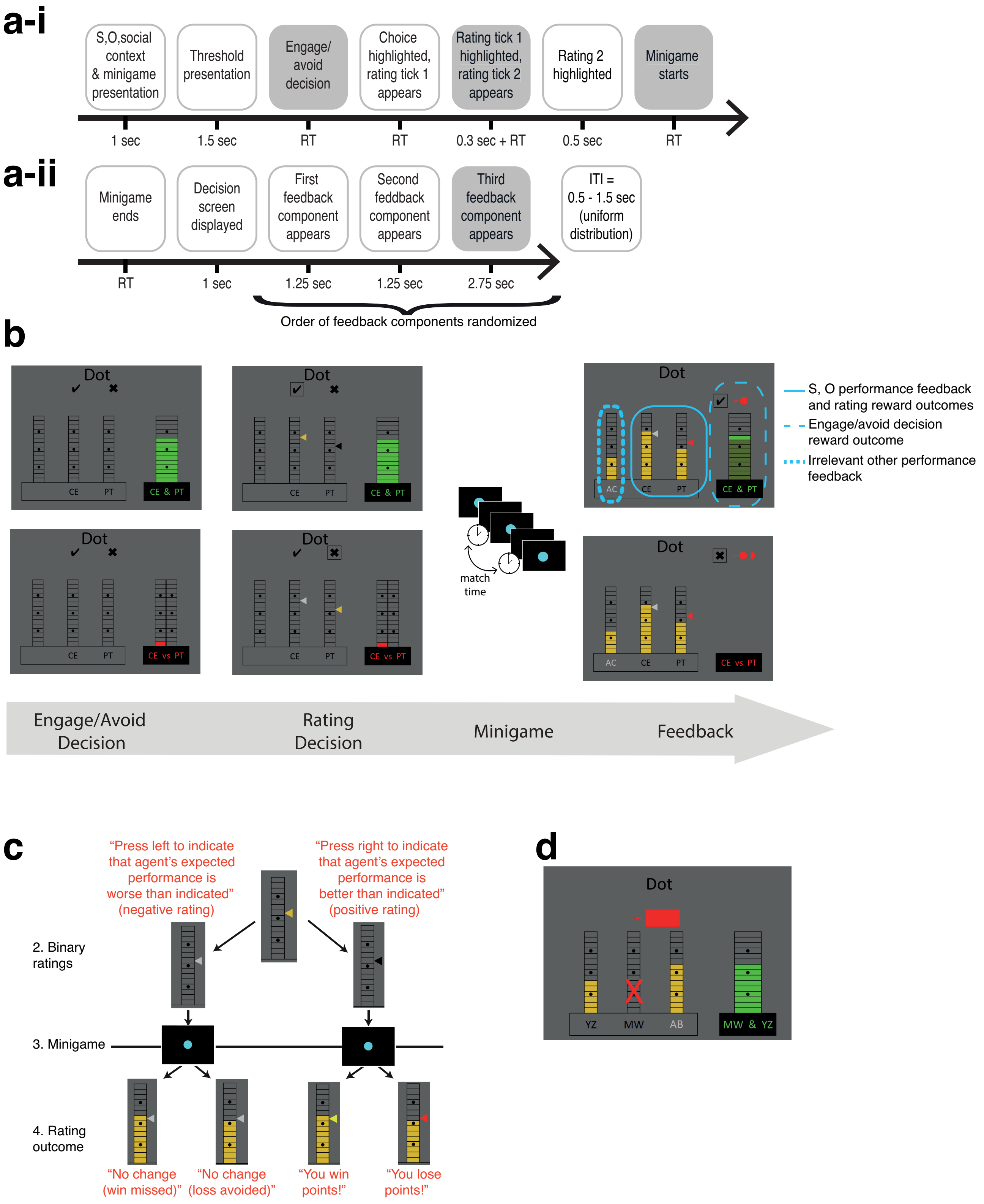

Figure S2. Trial structure, key events, rating rationale, and false starts. Related to Figure 1. (a) All events within a trial and their associated timings are depicted. Note that timing of the events is shown underneath the arrow and RT (reaction time) means that the respective step only ends when the participant makes a

response. Events highlighted in grey are depicted in more detail in (b) as they represent key events within the trial: engage/avoid decision, the rating decision, playing of the minigame and the feedback phase. The first part of a trial reveals the social context (competition or cooperation), participants then made an engage/avoid decision, and provided self (S) and relevant other (O) ratings **(a-i).** The feedback phase shows performances of all players during the minigame and possible points gained or lost from engage/avoid decisions and rating decisions. **(a-ii)**. **(b)** For illustration purposes, the same performance feedback is shown in a cooperative context (upper rows) and a competitive context (lower rows). At the beginning of a trial S (initials of the participant, middle position), O (initials of one of the two other players relevant for the current trial, left or right position), social context (cooperation or competition) and type of upcoming minigame ("Dot" indicates the dot task; see supplementary figure 3) is presented. The initials of the irrelevant other player are not shown, and the social context is color-coded as green for cooperation and red for competition. These colors are also used to indicate the threshold of the current trial (thresholds are shown as bars on the very right). In these examples, the choices made (see highlighted tick and cross in the first panel) are to engage in cooperation and to avoid competition. The choices are arbitrarily picked for illustration here, but in the experiment the decision to engage in cooperation would indicate the expectation that S and O will, together, perform better than the threshold of 10; the avoidance of competition would indicate that the participant does not expect to perform at least one point better than the O. After the engage/avoid choices, binary ratings of S and O take place (second panel) in randomized order. Once a rating has been made, the tick turns from yellow to either grey or black depending on whether one expects the selected player to perform respectively better or worse compared to the level indicated by the tick. In these examples, O rating occurred first in the cooperation trial and S rating occurred first in the competition trial (ratings have already been made and are therefore shown in grey/black). These ratings indicate that the participant expects to perform worse than 10.5, while the O is expected to perform better than 8.5 (see panel (c) for more information on color coding of rating ticks). Note that these ratings are consistent with the engage/avoid choices shown in the two examples. In the feedback phase (third panel), the previous screen from before the minigame reappears (see a-ii). However, the right side of the screen showing the cooperation/competition threshold is occluded if a participant had chosen to avoid cooperation/competition in the previous decision phase of the trial. This means that in this example trial the threshold is only shown again in the cooperate trial (because the participant actually decided to cooperate) and not in the compete trial (because in this example trial the participant refrained from competing). In other words, the repeated presentation of the threshold is not a feature of the social context, but entirely a consequence of the engage/avoid choice made. Subsequently, three feedback components appear in randomized order to control for sequence effects (see legend on the right-hand side). In cooperation, the choice payoff is -1 (red coin above threshold), because the average performance is 9 while the threshold is 10. In competition, the choice payoff is -1.5 which is due to chance (payoff from avoid choices is +1.5 or -1.5 with a 50/50 probability) and independent of performance feedback. Note that the participant would have earned a payoff of 3 had the engage choice been taken (performance feedback difference of +4 minus threshold of +1). Note that, overall, the magnitude of the rating payoff is marginal compared to the engage/avoid choice payoff. **(c)** Rationale of binary ratings. Before the minigame, participants indicated for S and O either a positive or a negative rating reflecting the expectation that the player would surpass or fall below a given rating marker. The color change of the rating marker was indicative of the choice made; the rating marker turned black for a positive rating and grey for a negative rating. A positive rating led to a win or loss of 0.25 points depending on subsequent performance feedback. A negative rating led to no change in the points count independent of performance feedback. Therefore, making a correct negative rating was associated with a benefit of avoiding losing points while making a correct positive rating was associated with a benefit of winning 0.25 points. Note that in panel (a-ii), the performance and rating feedback indicate an incorrect negative rating (missed win) for S and an incorrect positive rating (loss) for O. Red text in quotes is taken from the participants' instructions where a similar illustration was used. **(d)** The screenshot shows the feedback screen of a false start trial. In false start trials, the true performance of the participant in the minigame was below a predetermined threshold for acceptable performances. False start trials were a case of veridical performance feedback to ensure that performance feedback in general was believable. Participants incurred a loss of 3 points (indicated by the red bar) on those trials and no points could be won by the decision or the ratings. The feedback phase in false start trials was not analyzed and no prediction error for S was calculated on those trials (reward prediction error and prediction error for others were calculated as normal).

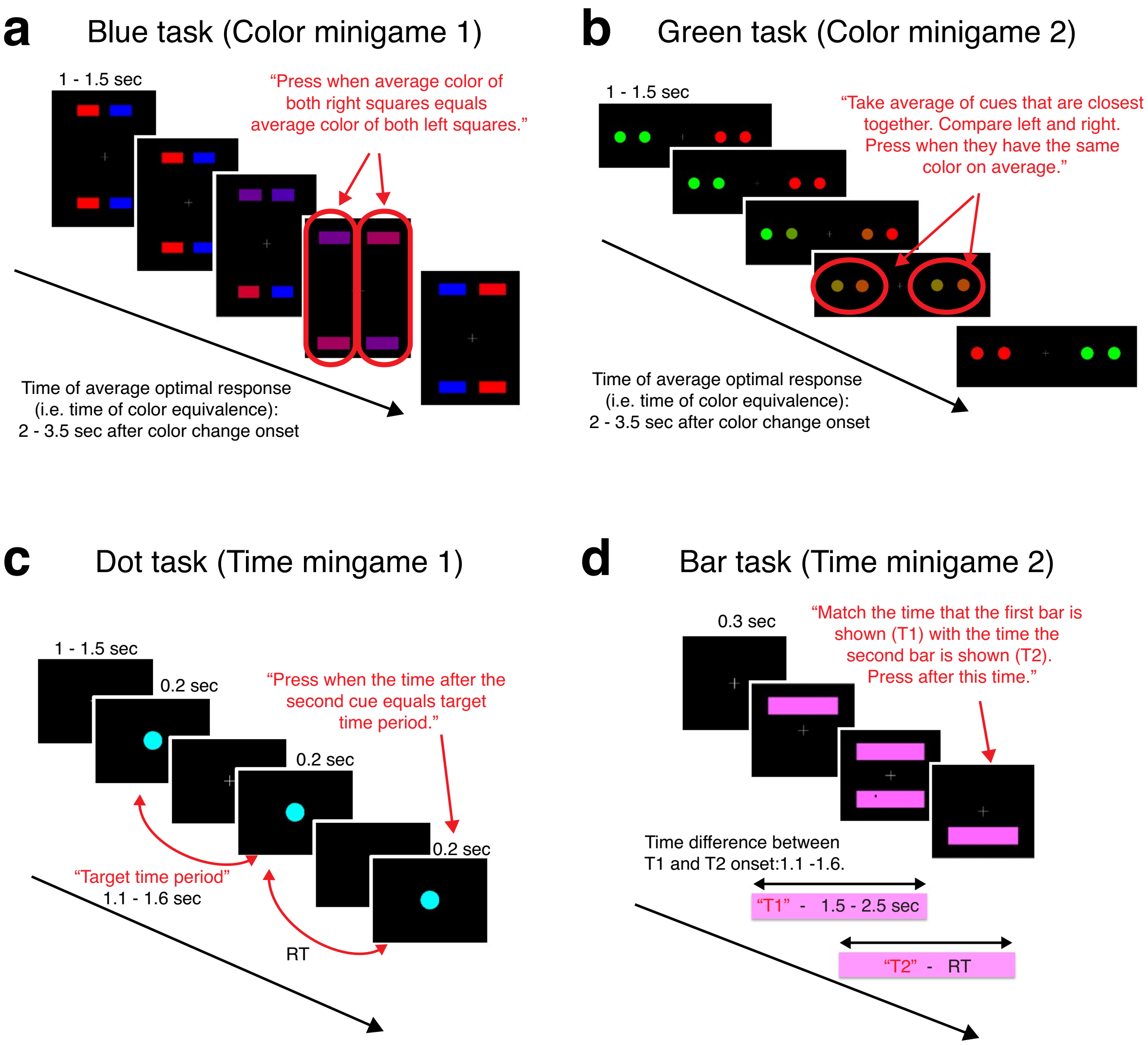

Figure S3. Minigame description. Related to Figure 1. We used two pairs of minigames in the experiment ('color minigames' and 'time minigames'), each assigned to one fMRI block. The order of the types of minigames was counter-balanced across participants (color minigames were assigned to the first session for one half of participants and to the second session for the other half) and this was orthogonal to the application of TMS. The minigames were necessary prerequisites for administering performance feedback on every trial, which was in fact pre-determined according to a finely balanced and decorrelated schedule. However, exceptions from this pre-determined schedule existed and constituted cases of veridical performance feedback; this was an additional measure to ensure that performance feedback was believable. These exceptions were 'false start' trials, in which the true performance was worse than a given performance threshold, and this then led to a feedback penalty (see section *Experimental Paradigm* of the supplementary text document). Minigames were short reaction-time based tasks and we designed them to be relatively non-transparent in the sense that the experience of performing the minigames was not very informative for estimating one's ability compared to the explicit performance feedback that was given to the participants. Also, we varied the timing parameters of the minigames to make it harder to compare true performance across different trials of a minigame and to ensure a temporal jitter between decision and outcome phases. The two time minigames were relatively similar to each other (participants estimated a target time period) and so were the two color minigames (participants estimated color equivalences). In consequence, the two fMRI sessions were framed to the participants as measuring very different aspects of cognitive performance. Generating this impression was important, because we used the same performance schedule for both fMRI sessions (see *Experimental task* section in Methods; Supplementary Fig.2). This meant that we could investigate and compare the behavioral and neural mechanisms underlying learning with the same performance schedule both with and without the application of cTBS. The framing in terms of very different types of minigames prevented participants from noticing this similarity and in fact none of the participants

expressed any suspicion spontaneously or after being questioned. Each minigame was performed with one response button press with the right index finger and the approximate time for each of the four minigames was similar (see figure for timing parameters). As in our previous study, participants performed all four tasks very well(Wittmann et al., 2016). We calculated an index of true performance in the tasks in the same way as in our previous work(Wittmann et al., 2016). However, this performance index was not used further when analyzing behavioral data; it was only used to determine thresholds for the above-mentioned 'false start' trials (see *Experimental task* section in Methods; Supplementary Fig.2), which was based on pilot experiments. Note again, that except for false start trials, the performance feedback was unrelated to this true performance measure. Note that the red text in the panels was used to explain the task to participants, who saw very similar illustrations of the task in the instructions. Black text supplements timing information. All varying timings were picked from a uniform distribution. **(a,b)** In the color minigames, two pairs of cues appeared on the screen and after an initial stable period, they began to change color until the colors fully reversed. The two color minigames, the blue task (panel a) and the green task (panel b), differed with respect to the colors used and the spatial organization of the colored cues on the screen. The underlying principle was the same however: Participants indicated with a button press when the colors of two of the cues reached the same average color as the two other cues that changed color in the opposite direction (red boxes indicate which cues were compared). This point of true color equivalence was reached after 2 – 3.5 seconds. **(c,d)** In the two time minigames, participants replicated a given time interval with the goal of matching a target time. In the dot task, this target time is the time between two blue dots that appear briefly on the screen in sequence. In the bar task, the target time is the time that a bar is initially presented on the screen and the goal is to replicate the target time for a second bar that occurs on the screen after a short delay. Target times were 1.1 – 1.6 seconds for the dot task and 1.5 – 2.5 seconds for the bar task. Although these target times were different, the overall time spent on both minigames was similar.

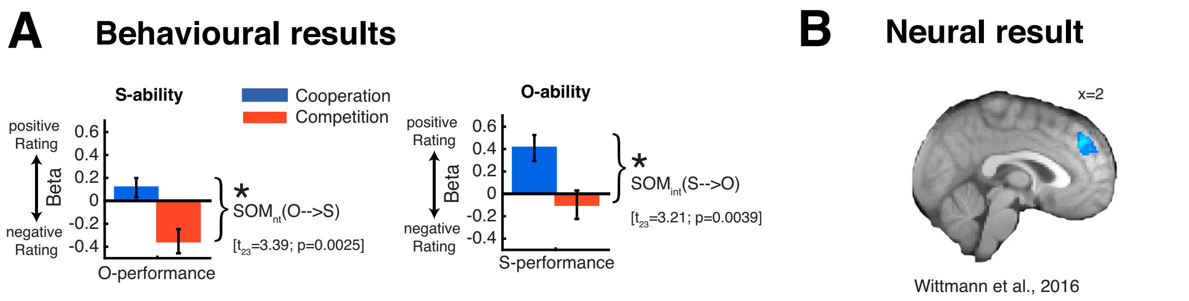

Figure S4. Results of previous report of self-other-mergence (Wittmann et al., 2016). Related to Figure 3. **(A)** Previously, we applied the same statistical analyses to the rating data for S-ability and O-ability and found significant SOM_{int} in both cases (blue = cooperative trials, red = competitive trials). Note that, as done here, significance testing was performed on the full GLM as reported in Supplementary Fig.5A. **(B)** Previously, we identified dmPFC area 9 using the contrast O-performance (shown in blue). In ROI analyses performed on the peak coordinates of this contrast, we found a significant neural SOM_{int}(S-->O) effect that correlated with the behavioral neural SOM_{int}(S-->O) effect. Therefore, we used the peak coordinates of the contrast shown in panel B as the target location for our dmPFC-cTBS group. Data are represented as mean ± SEM.

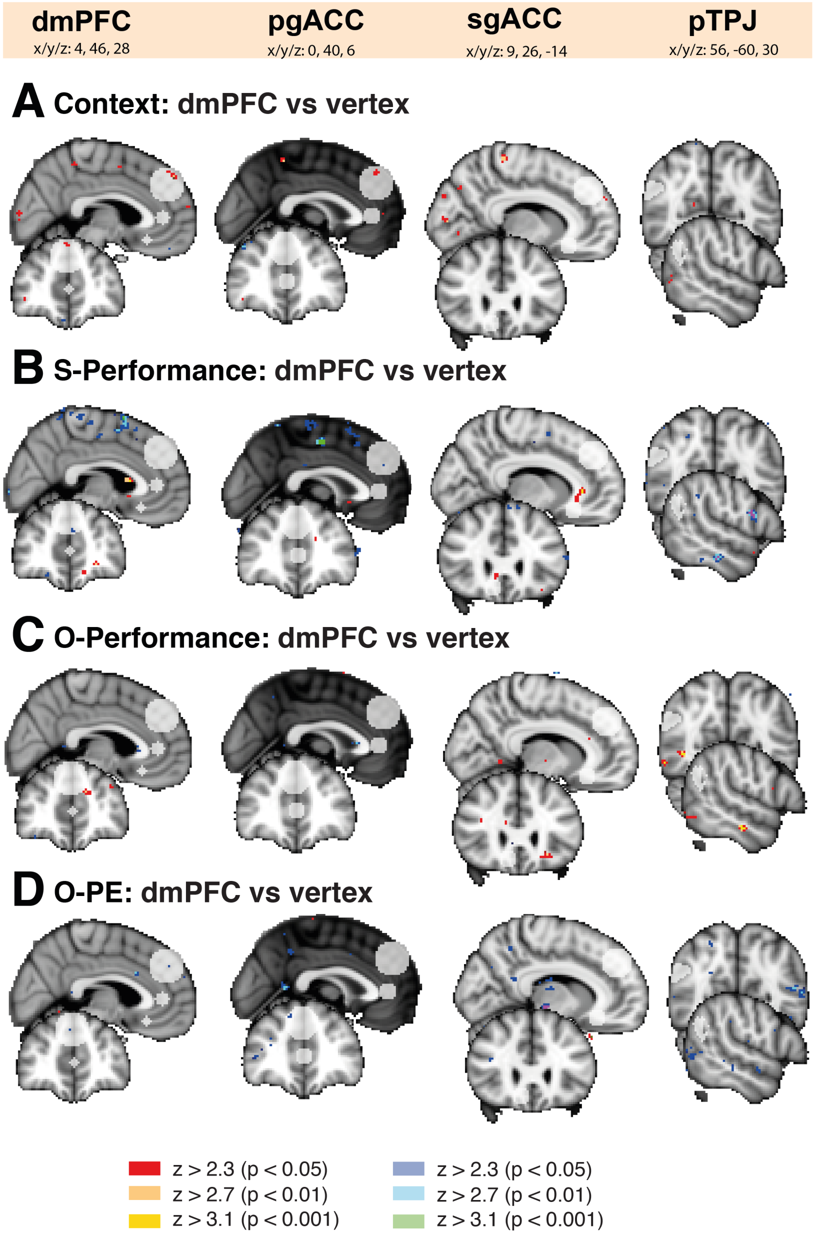

Figure S5. Additional neural effects of cTBS including sub-threshold activations. Related to Figures 5 and 6. Brain-wide effects are shown for the contrasts related to Fig.3A,B. Colors present uncorrected z-maps thresholded at z>3.1, z>2.7 and z>2.3 (red and blue represents respectively positive and negative activation). For transparency, we are showing additional variables that might potentially have been affected by dmPFC stimulation. As in Fig.6 we overlay additional ROIs in areas relevant to social cognition (pgACC, sgACC and pTPJ; see Fig.6 and main text for details). Modulation of activation is shown as a difference between cTBS and no-cTBS sessions in the dmPFC group compared to the control group. We do not observe modulation of activation as a result of stimulation (neither whole-brain nor in the ROIs) for **(A)** context (cooperation=1, competition=-1), **(B)** S-performance (performance history of Self), **(C)** O-performance (performance history of Other), or **(D)** O-PE (Prediction error associated with other's performance). These results show that dmPFC stimulation was specific to the SOM_{int}(S-->O) effects reported in the main text. In relation to the S-performance effect shown in panel B and our finding that S-performance was significantly changed during competition, we considered whether the S-performance effect in competition was related to the strength of SOM. However, there was no correlation between the neural effect of S-performance in no-cTBS during competition (collapsed over both groups; ROI taken from Fig.5C) and SOM_{int}(S-->O) (r= -0.140; p = 0.302) during no-cTBS.

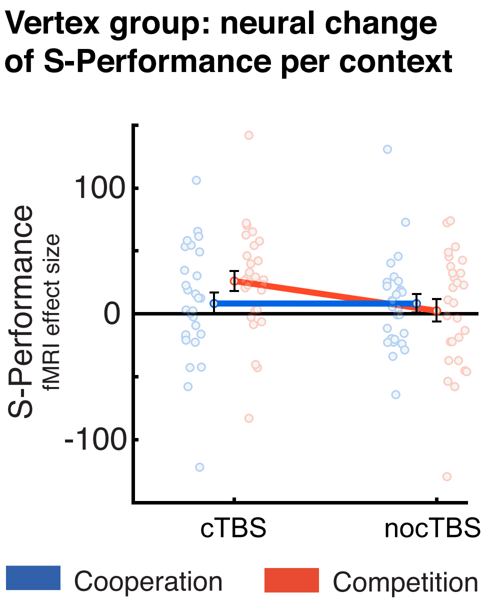

Supplementary Figure 6. Neural effects of S-performance in the vertex control group. Related to Figures 5 and 6. The panel shows the same regressor as main text Fig.5D, performed on fMRI data from the same ROI as in Fig.5D, but for the vertex control group. Data are represented as mean ± SEM.

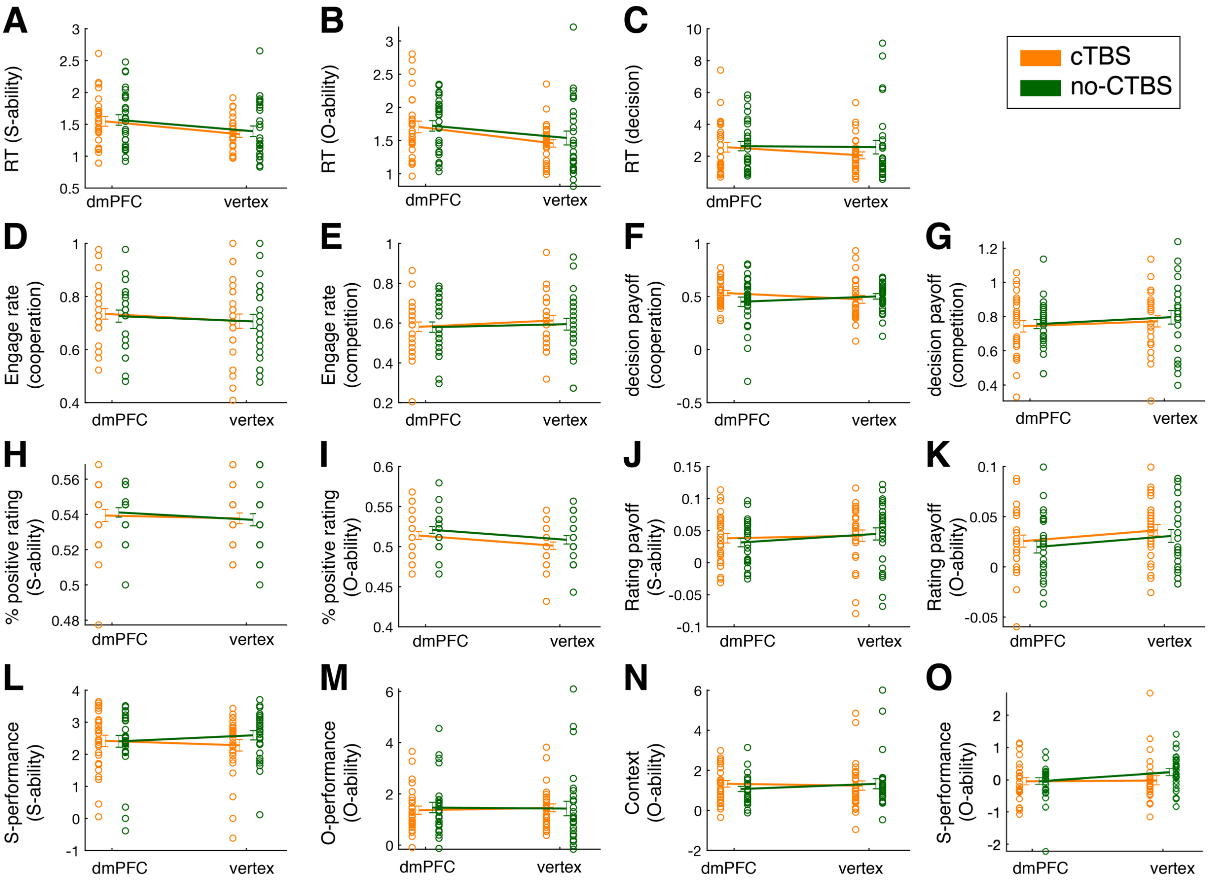

Figure S7. cTBS over dmPFC does not impact general task performance. Related to Figures 7. We conducted several behavioral control analyses that targeted different task-related variables that might change as a function of the application of cTBS over dmPFC. As for our critical statistical test of interest, the cTBS induced change of SOM_{int}(S-->O), we calculated a mixed-effects analysis of variance to test for a dmPFC/vertex by cTBS/no-cTBS interaction effect. Despite considering a broad range of task variables, we found no significant interaction effects, suggesting that our causal manipulation targeting dmPFC specifically impacted SOM_{int}(S-->O). **(A,B,C)** First, we considered reaction times related to the S-ability and O-ability ratings as well as to the engage/avoid decision. Such reaction time effects might reveal broad changes in the time necessary to compute information for oneself, the other player, or to combine this information flexibly towards a decision. Hence, analyses of reaction time might reveal specific deficits in one or several of these domains. We found no significant interaction effect either for the reaction times for S-ability-rating ($F(1,54) = 0.042$, $p = 0.839$), O-ability-rating $F(1,54) = 0.260$, $p = 0.612$), or for the engage/avoid decision $F(1,54) = 1.670$, $p = 0.202$. **(D,E,F,G)** Next, we considered whether the general ability to make decisions in cooperative or competitive contexts was impacted by the application of cTBS. Decisions require an integration of self-and other-related performance estimates and might be impacted differentially based on whether the context required participants to combine performance estimates during cooperation or to contrast performance estimates during competition. Therefore, we examined whether the frequency of making an engage choice was altered by the application of cTBS over dmPFC. There were no significant interaction effects for the rate of engage choices either in cooperation ($F(1,54) = 0.062$, $p = 0.804$), or in competition ($F(1,54) = 0.334$, $p = 0.566$). In addition, the payoff that resulted from these decisions did not change as a function of the cTBS application either in cooperation ($F(1,54) = 2.872$, $p = 0.096$) or competition ($F(1,54) = 0.030$, $p = 0.863$). **(H,I,J,K)** After this, we considered whether stimulating dmPFC might have affected performance estimation for one of the players in some more general way as opposed to in a manner that was specifically related to self-other-mergence. We compared the rate at which a player was estimated as better than indicated by the rating tick (see Supplementary Fig.2 for details of the rationale behind the ability rating). However, this measure of rating performance was not impacted by the cTBS stimulation either for S-ability ratings ($F(1,54) = 0.339$, $p = 0.563$) or for O-ability ratings ($F(1,54) = 0.001$, $p = 0.985$). In addition, the payoff received for S-ability ratings ($F(1,54) = 1.121$, $p = 0.294$) and O-ability ratings ($F(1,54) = 0.001$, $p = 0.979$) was not significantly impacted by the cTBS. The absence of such general performance impairments further suggests that our causal manipulation impacted a very specific aspect of self and other-related performance estimation. **(L,M,N)** We went on to perform an even more

sensitive test to assess whether cTBS over dmPFC induced deficits in “appropriate” S-ability or O-ability estimation – the degree to which ability estimates reflect performance by the same person (i.e. appropriate estimates of O-ability should be based on O-performance and appropriate estimates of S-ability should be based on S-performance). Specifically, we extracted the S-performance effect from the S-ability GLM and compared it across stimulation conditions and groups. We performed an analogous comparison for the O-performance effects in the O-ability GLM. Both of these effects assess how much estimates of ability are based on the previous history of performance of the same player. We believe that this is the most sensitive measure of appropriate ability performance learning in our current set of analyses. We performed this test by comparing effect sizes from our main GLMs of interest predicting S-ability and O-ability (Figs.4,7). Note that we used the variance-weighted beta weights (Matlab’s stats.t object) for the S-ability GLM to approximate the effect sizes because of difficulties in estimate effect sizes for all participants as described in the methods. The O-ability GLM employed standard beta weights as proxies for the effect sizes. However, we found no cTBS induced changes in appropriate ability estimation either for S-ability ($F(1,54) = 1.422$, $p = 0.238$) or for O-ability ($F(1,54) = 0.094$, $p = 0.760$). **(N)** Next, we considered whether cTBS over dmPFC might have affect a more non-specific bias in the estimation of the other player’s performance. As we have shown in Fig.4 (see main text), participants showed an other-specific optimism bias in performance estimation. They evaluated the other player more positively in cooperation than during competition. Importantly, this bias is present in addition to SOM_{int} and, in contrast to SOM_{int}, it is unrelated to the specific levels of performance of the irrelevant player. However, the optimism bias in performance estimation (*Context* effect in O-ability-ratings) was not altered by the cTBS ($F(1,54) = 0.975$, $p = 0.328$). Together, these results suggest that cTBS over dmPFC specifically altered only SOM_{int}(S-->O). It did not affect more general measure of task performance including choice frequencies and payoffs during decision, Self-rating and Other-rating. Neither did it induce a general deficit in performance tracking for one of the agents, or changed the optimism bias during the O-ability estimation. Instead, as shown in Fig.7, cTBS over dmPFC specifically affected the degree to which O-ability was referenced to one’s own performance in a context-dependent manner. **(O)** Finally, we considered whether cTBS might have impacted on S-performance during the estimation of O-ability. S-performance is tightly related to our key effect of interest, SOM_{int}(S-->O), which is the effect of “S-performance x Context” in the estimation of O-ability. Significant effects of S-performance here indicate that cTBS-induced changes in SOM occur mostly in one of the two social contexts, cooperation or competition. However, we did not find a significant main effect of S-performance on O-ability estimation ($F(1,54) = 1.712$, $p = 0.196$) suggesting that dmPFC cTBS does not result in uniformly increased or decreased effects of S-performance on O-ability and therefore also does not suggest that cTBS-induced changes on SOMint(S-->O) are mainly driven by one of the two social contexts. Data are represented as mean $\pm$ SEM.

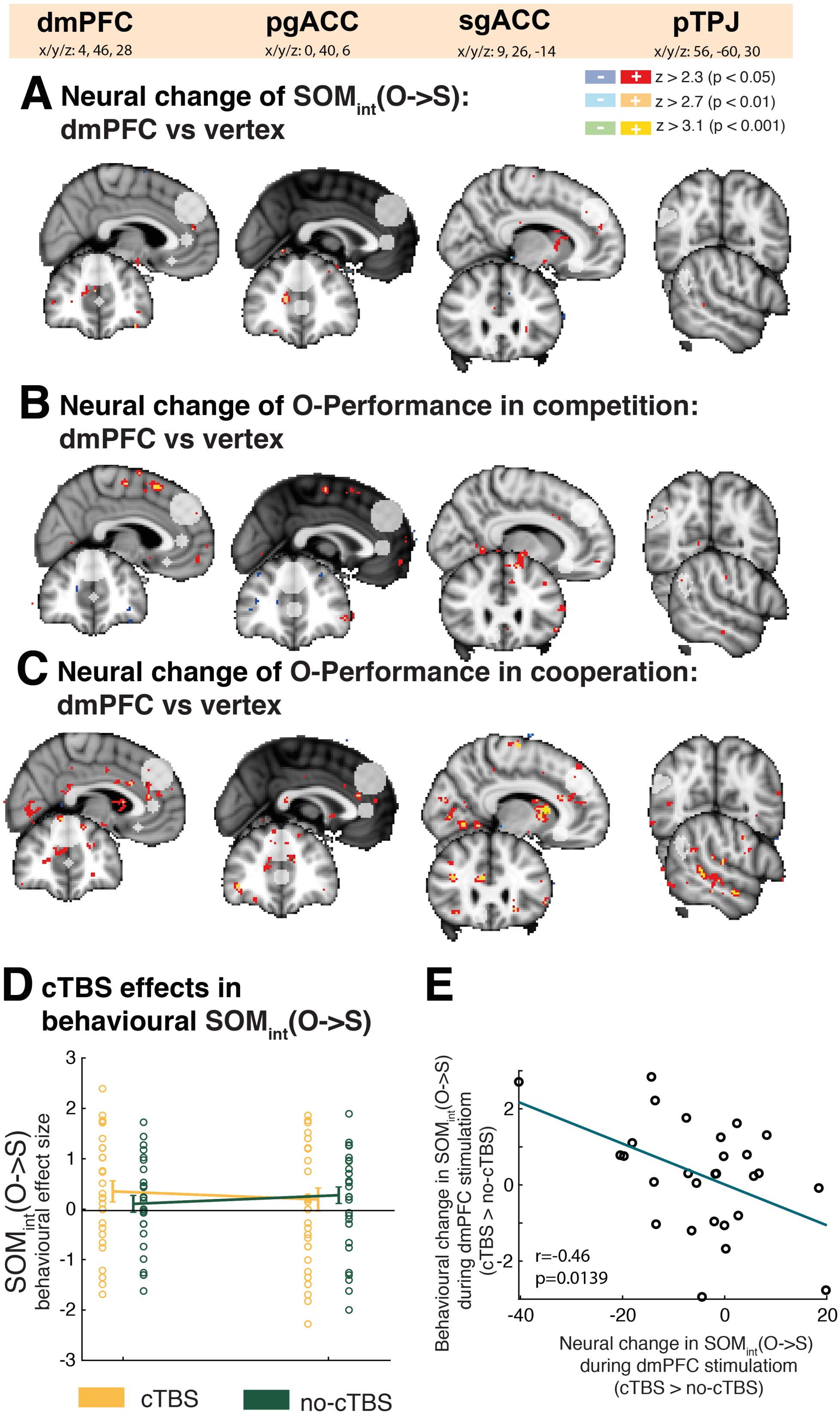

Figure S8. cTBS effects on SOM_{int}(O-->S). Related to Figure 5,6 and 7. In our previous work (Wittmann et al., 2016), we found that signals in dmPFC were correlated with self-other-mergence in multiple ways suggesting that it encodes reciprocal influences of our own performance estimates on estimates of another person and vice versa. However, of the two self-other-mergence effects we discovered in behavior, only SOM_{int}(S-->O) was directly correlated with neural activity in dmPFC. For this reason, in the main text, we focus on this direction of

influence instead of SOM_{int}(O-->S). To complement our results in the main text, in this supplementary figure, we examine the effects of cTBS on SOM_{int}(O-->S) following the same analysis steps as for our main analyses. **(A,B,C)** These panels show a series of whole brain analyses examining SOM_{int}(O-->S) related effects in the same region of interest in dmPFC. As in Fig.6 in the main text we overlay additional ROIs in areas relevant to social cognition (pgACC, sgACC and pTPJ; see Fig.6 and main text for details). We considered whether cTBS might have an effect on SOM_{int}(O-->S) (i.e. the variable *O-performance x Context,* see Methods; panel A). In addition, as we had observed that the effects of cTBS on SOM_{int}(S-->O) were particularly pronounced in the competition condition, we also separated SOM_{int}(O-->S) into its component parts: *O-performance* during competitive trials (panel B) and *O-performance* in cooperative trials (panel C). For all three contrast, we assessed cTBS-induced neural effects as the difference between cTBS and no-cTBS condition for dmPFC stimulation compared to the same difference for vertex control group. This stringent comparison ensured that we could isolate neural effects that specifically emerged as a consequence of causally manipulating activity in dmPFC. More formally, the contrasts show the effects of "dmPFC (cTBS – no-cTBS) > vertex(cTBS – no-cTBS)" for all three variables of interest. However, we found no ROI corrected or whole-brain corrected results for any of the three contrasts neither in dmPFC nor in any of the other social cognition-related ROIs. Effects are shown at uncorrected threshold levels for illustration (blue and red colors respectively, represent negative and positive activations). **(D)** Despite the absence of neural changes in SOM_{int}(O-->S), we examined whether the cTBS on dmPFC might have altered the behavioral effect of SOM_{int}(O-->S). We used the complementary GLM to the one for SOM_{int}(S-->O) (see main text and Fig.4, Methods) and examined the *O-performance x Context* variable. We used the t-stats for inference (see explanation in main text and Fig.4). We predicted that cTBS on dmPFC should have a similar effect on SOM_{int}(O-->S) as it had for SOM_{int}(S-->O), namely an increase in self-other-mergence after disruption of dmPFC activity with cTBS. Although the direction of effects that we found is consistent with this hypothesis, the relevant interaction between group (dmPFC/vertex) and stimulation (cTBS/no-cTBS) is not significant (2-way mixed effects ANOVA ($F(1,54) = 0.976$, $p = 0.328$). Therefore, consistent with the absence of neural changes in SOM_{int}(O-->S), we also did not find behavioral changes in SOM_{int}(O-->S) induced by cTBS over dmpFC. **(F)** Finally, in the absence of changes in neural or behavioral SOM_{int}(O-->S) in our sample as a whole, we considered the possibility that cTBS might have disrupted the neural representation of SOM_{int}(O-->S) more in some participants than in others and that these participants might in consequence display increased behavioral SOM_{int}(O-->S). We used a cluster-shaped mask of the effect shown in Fig.5B as region of interest, because the corresponding neural changes were the strongest cTBS induced effect in our data set. We reasoned that cTBS might have the strongest effects on SOM_{int}(O-->S) in this part of the brain. Note that this correlation analysis is statistically independent of the ROI selection because it examines individual variation in a variable unrelated to the ROI selection. From this ROI, we extracted the second level contrast maps (cTBS > no-cTBS) (see Methods for more information on the fMRI analysis pipeline). Indeed, we found a correlation between the neural change of SOM_{int}(O-->S) in the dmPFC group and their change in behavioral SOM_{int}(O-->S): Participants with a stronger decrease in neural SOM_{int}(O-->S) exhibited a stronger increase in behavioral SOM_{int}(O-->S) ($r=-0.46$; $p=0.0139$). In summary, these results show that both neural and behavioral SOM_{int}(O-->S) are not as strongly impacted as SOM_{int}(S-->O) by the application of cTBS over dmPFC. However, the correlation between neural and behavioral SOM_{int}(O-->S) effects across participants suggests that dmPFC carries information about self-other-mergence also in the direction other-to-self (Wittmann et al., 2016) – variation in neural signals predict the degree by which individuals are influenced by others in their self-assessments. Our results suggest that dmPFC is part of a wider neural network computing the relationships between self and other performance in both directions of influence. However, in contrast to the causal importance of dmPFC for SOM_{int}(S-->O), SOM_{int}(O-->S) might be controlled by a tightly connected brain region that shares information about the SOM_{int}(O-->S) with dmPFC. Data are represented as mean $\pm$ SEM.
